# GSK3βhigh/NFATc1high subtype targeting overcomes therapy resistance in pancreatic cancer through transcriptional induction of homologous recombination repair

**DOI:** 10.1136/gutjnl-2025-336227

**Published:** 2025-12-31

**Authors:** Muhammad Umair Latif, Xueang Liu, Aiko Bockelmann, Laura Huhnold, Geske Elisabeth Schmidt, Lukas Klein, Xueyuan Zhao, Lena-Christin Conradi, Karly Conrads, Anna Lena Weber, Sercan Mercan, Kristina Reutlinger, Atmika Paul, Zeynab Najafova, Steven A Johnsen, Zuriñe Bonilla Del Rio, Frederike Penz, Jovan Todorovic, Holger Bastians, Tim Beissbarth, Ulrich Sax, Ramy Ashry, Oliver H Krämer, Elisabeth Hessmann, Günter Schneider, Philipp Stroebel, Ivan Bogeski, Shiv K Singh, Volker Ellenrieder

**Affiliations:** 1 Department of Gastroenterology, Gastrointestinal Oncology and Endocrinology, University Medical Center Göttingen, Göttingen, Lower Saxony, Germany; 2 Clinical Research Unit 5002, KFO5002, University Medical Center Göttingen, Göttingen, Lower Saxony, Germany; 3 General and Visceral Surgery, University Medical Center Göttingen, Göttingen, Lower Saxony, Germany; 4 Department of Medical Bioinformatics, University Medical Center Göttingen, Göttingen, Lower Saxony, Germany; 5 Department for Molecular Oncology, University Medical Center Göttingen, Göttingen, Lower Saxony, Germany; 6 Robert Bosch Centre for Tumour Diseases, Stuttgart, BW, Germany; 7 Institute of Pathology, University Medical Center Göttingen, Göttingen, NDS, Germany; 8 Department of Medical Informatics, University Medical Center Göttingen, Göttingen, NDS, Germany; 9 Institute of Toxicology, University Medical Center Mainz, Mainz, Germany; 10 Department of Oral Pathology, Faculty of Dentistry, Mansoura University, Mansoura, Egypt, Al-Mansura, Egypt; 11 Clinic of General, Visceral and Pediatric Surgery, University Medical Center Göttingen, Göttingen, Lower Saxony, Germany; 12 Molecular Physiology, Institute of Cardiovascular Physiology, University Medical Center Göttingen, Gottingen, Niedersachsen, Germany

**Keywords:** DNA DAMAGE, PANCREATIC CANCER, DRUG RESISTANCE, CELL DEATH, CANCER

## Abstract

**Background:**

The efficacy of pharmacological glycogen synthase kinase-3β (GSK3β) inhibition is currently being investigated in unselected cohorts of metastatic pancreatic ductal adenocarcinoma (PDAC). Here, we sought to determine the clinical significance of nuclear GSK3β accumulation in patients with resectable PDAC.

**Objective:**

This study aimed to explore the therapeutic potential and underlying mechanisms of GSK3β pathway disruption in PDAC with enriched nuclear GSK3β levels.

**Design:**

We investigated the activation and function of GSK3β and its downstream transcription factor NFATc1 in tumour recurrence, growth and resistance using human PDAC tissues, patient-derived organoids and tumour cells, PDAC explants, cell lines and murine models. GSK3β signalling was disrupted using genetic and pharmacological approaches. Live-cell imaging, proliferation, homologous recombination (HR) repair and comet assays, messenger RNA sequencing and chromatin immunoprecipitation were used to explore GSK3β-NFATc1 signalling-mediated target gene regulation in DNA repair, growth and resistance.

**Results:**

Nuclear GSK3β accumulates in a subset of resected PDAC and promotes proliferation and DNA repair through NFATc1. The GSK3β^high^/NFATc1^high^ subtype accounts for 14% of resected PDAC and is associated with rapid tumour recurrence and poor survival. The GSK3β-NFATc1 signalling pathway contributes to cisplatin resistance by inducing BRCA genes transcription, which facilitates HR-mediated DNA double-strand breaks (DSBs) repair. Disruption of the GSK3β-NFATc1 axis impairs HR-driven DSB repair, increasing cisplatin sensitivity in vitro and in preclinical PDAC models.

**Conclusion:**

We have identified a highly aggressive GSK3β^high^/NFATc1^high^ subtype that predicts early recurrence, poor survival and cisplatin resistance in PDAC. This subtype reveals new treatment vulnerabilities suggesting that patients with PDAC may benefit from stratification-based tailored treatment strategies.

**WHAT IS ALREADY KNOWN ON THIS TOPIC**

- Pancreatic ductal adenocarcinoma (PDAC) can be classified into different molecular subtypes with largely unclear prognostic and therapeutic relevance.
- PDAC with homologous recombination (HR) repair defects shows increased sensitivity to platinum-based therapy.
- Nuclear glycogen synthase kinase-3β (GSK3β) accumulation is associated with the acquisition of a resistant phenotype in PDAC.
- Nuclear GSK3β cooperates with and stabilises NFAT from proteasomal degradation in PDAC.

**WHAT THIS STUDY ADDS**

- Nuclear accumulation of GSK3β-NFATc1 signalling in tumour cells defines a novel highly aggressive (GSK3β^high^/NFATc1^high^) PDAC subtype, predicting early recurrence and poor survival after surgery.
- Nuclear GSK3β promotes growth and DNA repair by activating NFATc1-dependent transcription of HR repair genes, such as RAD51, BRCA1, BRCA2 and FANC family members.
- Inactivation of nuclear GSK3β-NFATc1 signalling impairs HR and enhances efficacy of cisplatin in GSK3β^high^/NFATc1^high^ PDAC.

**HOW THIS STUDY MIGHT AFFECT RESEARCH, PRACTICE OR POLICY**

- This study demonstrates a strong correlation between the GSK3β^high^/NFATc1^high^ subtype and rapid tumour recurrence, as well as short-term survival in patients with resected PDAC.
- Future research is needed to validate these findings in larger patient cohorts and to guide future treatment decisions.
- Additionally, stratification-based clinical trials are necessary to determine whether pharmacological inhibition of the GSK3β-NFATc1 axis in GSK3β^high^/NFATc1^high^ subtype tumours can improve the efficacy of platinum-based chemotherapy in PDAC.

## Introduction

 The prognosis of pancreatic ductal adenocarcinoma (PDAC) remains dismal.^1 2^ The main causes for the poor 5-year survival rate of only 10–12% include aggressive tumour growth, the high recurrence rate after surgical resection and the remarkable resistance to chemotherapy.^2–4^ At the time of diagnosis, only 15–20% of patients with PDAC present with a primarily resectable tumour, and despite complete oncological resection (R0) followed by adjuvant chemotherapy with platinum-based regimens (eg, FOLFIRINOX), the risk of disease recurrence remains high. Established risk factors for early tumour recurrence are, above all, incomplete oncological resection (R1), high-grade histology and delayed or incomplete use of adjuvant chemotherapy.^3 5–7^ In addition to these well-known criteria, growing evidence indicates that local immune regulation and in particular tumour subtype-specific molecular traits significantly impact on PDAC recurrence and resistance to chemotherapy.^8–11^ Identifying clinically relevant molecular and genetic abnormalities could therefore not only enhance our understanding of subtype-specific tumour behaviour but also lead to the development of novel treatment strategies for PDAC.^8–10 12 13^ However, scientific efforts are currently focusing on limited genetic alterations, such as oncogenic KRAS^G12C^ and KRAS^G12D^ mutations, BRAF, NTRK and tumours with microsatellite instability (MSI^high^).^10 14 15^ Of particular therapeutic relevance, however, are tumours with somatic or germline mutations of BRCA1, BRCA2 and other genes involved in homologous recombination (HR) repair.^14 16–18^ As such, tumours with mutations in HR genes often display increased susceptibility to platinum-based therapy and PARP inhibition.^14 19 20^ Accordingly, maintenance therapy with PARP inhibitors is recommended in patients with germline mutations of either BRCA1 or BRCA2 and confirmed stable disease under platinum-containing first-line therapy.^14 19–22^ Functionally, HR genes are crucial for error-free repair of DNA double-strand breaks (DSBs),^19–21^ and not surprisingly, activation of the HR system fosters the development of resistance against platinum (eg, cisplatin and oxaliplatin) and other DSB-inducing drugs (eg, irinotecan or SN38). Therefore, identifying pathways that trigger HR-mediated DSB repair will not only improve our understanding of treatment failures but may also open the avenues for novel treatment strategies to overcome platinum-based therapy resistance. In this context, signalling through the glycogen synthase kinase-3β (GSK3β) serine/threonine protein kinase is particularly interesting, as recent studies revealed a prominent role of the kinase in the acquisition of a highly aggressive and resistant phenotype in PDAC.^23 24^ Phase 1/2 clinical (NCT05077800, NCT05239182) trials are currently underway in patients with advanced PDAC to test the efficacy and tolerability of specific GSK3β inhibitors (GSK3β-i) such as 9-ING-41, in combination with chemotherapy. Although there is considerable evidence of tumour-promoting functions of GSK3β in PDAC and other cancers (eg, colorectal cancer and glioblastoma), it is worth noting that the kinase may also exert antitumour functions.^24–27^ Collectively, it appears likely that the dual functions of GSK3β in cancer are largely dependent on the cellular localisation, with oncogenic functions driven by nuclear accumulation of the kinase.^23 28–30^ Here, we examined the expression, tumour cell localisation and clinical impact of GSK3β signalling in resectable PDAC, and in addition, explored in-depth its role in growth, DNA repair and resistance to platinum-based therapy. We identify a previously unrecognised PDAC subtype characterised by nuclear co-expression of GSK3β and the transcription factor NFATc1. The GSK3β^high^/NFATc1^high^ tumour subtype is associated with an aggressive PDAC phenotype, early tumour recurrence and poor survival rates. Functionally, we demonstrate that nuclear GSK3β-NFATc1 signalling promotes tumour growth and fosters resistance through transcriptional activation of HR and subsequent repair of DNA DSBs. Furthermore, we present preclinical evidence that tailored inhibition of GSK3β-NFATc1 signalling blocks HR-induced DNA repair and enhances the efficacy of platinum-based therapy in GSK3β^high^/NFATc1^high^ PDAC.

## Results

### Protumourigenic role of nuclear GSK3β in PDAC progression and growth

**Figure 1:**
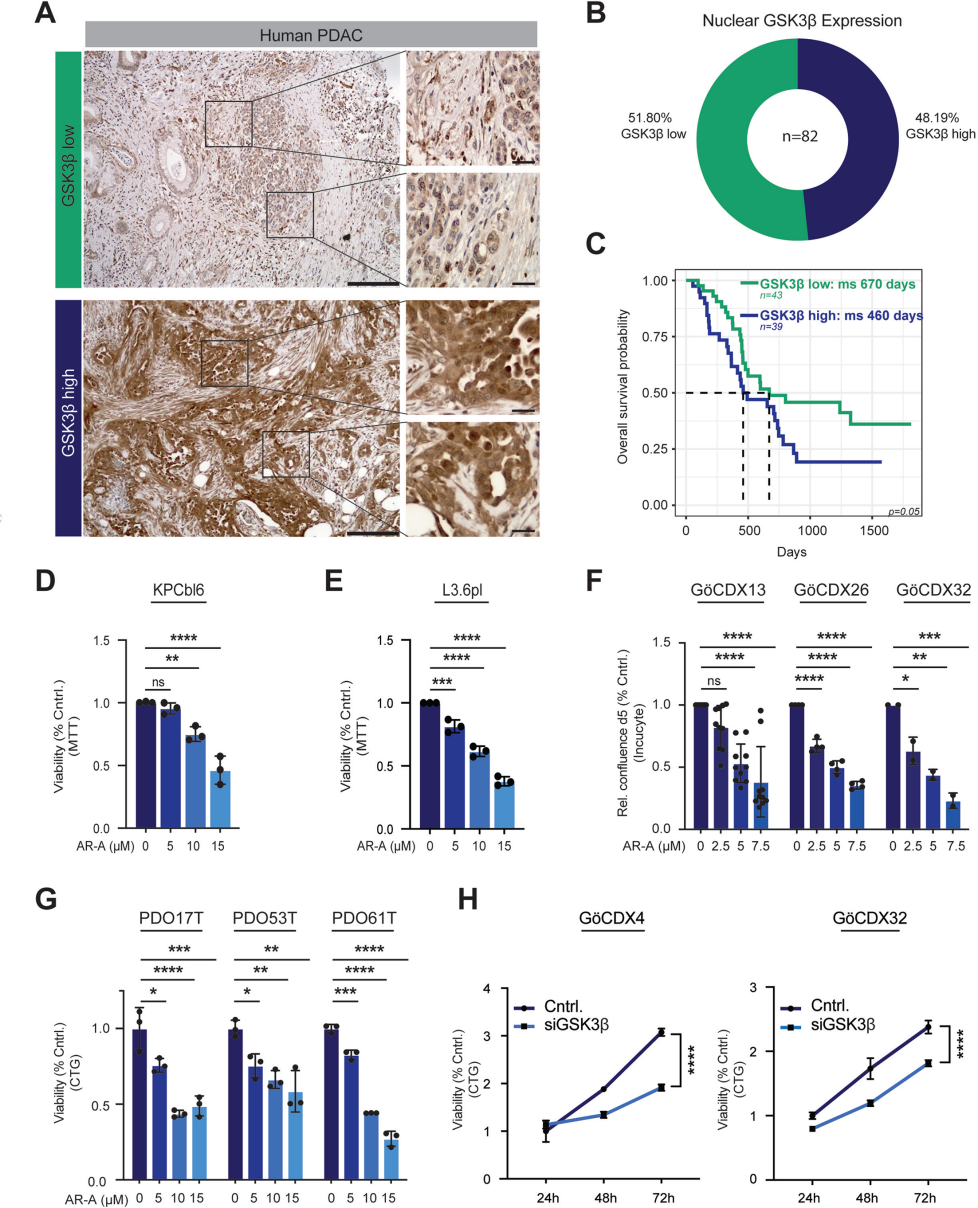
Nuclear GSK3β accumulation promotes PDAC growth. (**A**) Representative images of the GSK3β IHC, displaying GSK3β positive tumour cells in PDAC patient tissues. Scale bar indicates 200 µm and 100 µm. (**B**) Classification of patients with PDAC cohort into low and high GSK3β expression groups based on percentage of GSK3β nuclear positive tumour cells. n=82. (**C**) Kaplan-Meier plot of PDAC patients survival with low and high expression of nuclear GSK3β expression; n=82. (**D–E**) Cell viability in (**D**) KPCbl6 and (**E**) L3.6pl cells was analysed using MTT assay following treatment with increasing concentrations of AR-A (GSK3β-i) for 72 hours. (**F**) Primary tumour cell proliferation (GöCDX13, GöCDX26 and GöCDX32 cells) was determined by IncuCyte analyses following exposure to increasing concentrations of AR-A for 5 days. (**G**) Cell viability was assessed in PDOs using CTG-3D assay after treatment with increasing concentrations of AR-A for 72 hours. (**H**) Cell viability was determined in GöCDX4 and GöCDX32 cells by CTG analyses following introduction of siGSK3β for 24, 48 and 72 hours. Data are shown as mean±SD. Statistical analysis was performed using one-way ANOVA with Dunnett’s post-test, where *p≤0.05, **p≤0.01, ***p≤0.001, ****p≤0.0001. ANOVA, analysis of variance; CTG, CellTiter-Glo; GSK3β, glycogen synthase kinase-3β; GSK3β-i, GSK3β inhibitors; IHC, immunohistochemistry; MTT, 3-[4,5-dimethylthiazol-2-yl]-2,5 diphenyl tetrazolium bromide; PDAC, pancreatic ductal adenocarcinoma; PDOs, patient-derived organoids; siGSK3β, silencing of GSK3β.

The clinical and functional relevance of GSK3β was established using publicly accessible the cancer genome atlas program (TCGA), QCMG and Puleo *et al* data sets and in resected PDAC specimens from clinically annotated patients enrolled in the KFO5002 research programme at the University Medical Center Göttingen. In line with the suspected tumour-promoting activities of GSK3β, TCGA, QCMG and Puleo mRNA expression data from more than 560 patients with resected PDAC confirmed a significant correlation between high levels of GSK3β and poor patient survival after surgery (online supplemental figure S1A). Furthermore, immunohistochemistry (IHC) staining and subsequent QuPath analysis of 82 resected PDAC tumours from the KFO5002 cohort revealed low GSK3β nuclear abundance (ie, <45% of tumour cells positive; see online supplemental methods) in approximately half the patients (52%; figure 1A and B). However, GSK3β was found to be highly enriched in the nuclei of tumour cells (>45% of tumour cells positive) in 48% of resected PDAC cases (referred to as GSK3β^high^ tumours) (figure 1A,B). Additionally, nuclear accumulation of GSK3β correlated with increased kinase activity as indicated by the levels of Tyr-216 phosphorylation (online supplemental figure S1B and C) and predicted poor patient outcome, demonstrated by reduced relapse-free and overall survival (figure 1C and online supplemental figure S1D). In contrast, tumours with a primarily cytoplasmic localisation of GSK3β also exhibited lower kinase activity (as evidenced by reduced Tyr-216 phosphorylation levels) and tended to show a better patient survival (online supplemental figure S1E). We also investigated the expression and prognostic relevance of GSK3α kinase. GSK3α shares 85% sequence similarity with GSK3β, and previous studies have indicated both oncogenic and tumour suppressor properties in various tumour types.^31 32^ However, we did not find a significant correlation between GSK3α expression and patient survival neither in IHC/QuPath analysis of resected PDAC from the KFO5002 cohort (online supplemental figure S1F), nor in the cancer genome atlas program (TCGA) and human protein ATLAS database analyses (data not shown). Collectively, these results indicated a potential role for nuclear GSK3β in resectable PDAC and disease progression, prompting us to conduct a more detailed assessment of its oncogenic properties. To this end, we took advantage of various human and murine PDAC models, including primary cancer cells from genetically modified mice (eg, KPCbl6 cells), patient-derived primary cells (GöCDX cells) and organoids (PDOs) generated from resected PDAC of the KFO5002 cohort. Human and murine PDAC cells, GöCDX cells and PDOs were employed for viability and growth studies and either treated with specific GSK3-i, namely AR-A014418 (hereafter referred to as AR-A) and 9-ING-41 or transfected with siRNA against GSK3β to disrupt kinase signalling. The application of GSK3β-i resulted in robust, dose-dependent and time-dependent inhibition of cell viability (measured by MTT (3-[4,5-dimethylthiazol-2-yl]-2,5 diphenyl tetrazolium bromide) and CellTiter-Glo assays) and growth (determined by IncuCyte Live Cell imaging microscopy in all tested human and murine PDAC models with high GSK3β levels and irrespective of the genetic KRAS status (figure 1D–G; online supplemental figure S1G-I; online supplemental table S7). Similar effects were observed on RNA interference-mediated silencing of GSK3β (siGSK3β) (figure 1H). Collectively, these experiments confirmed the clinical relevance of nuclear GSK3β in resectable PDAC and suggested strong protumourigenic functions. In addition, these studies indicated that GSK3β specifically interferes with growth promotion in PDAC.

### GSK3β regulates homologous recombination repair gene signatures

**Figure 2:**
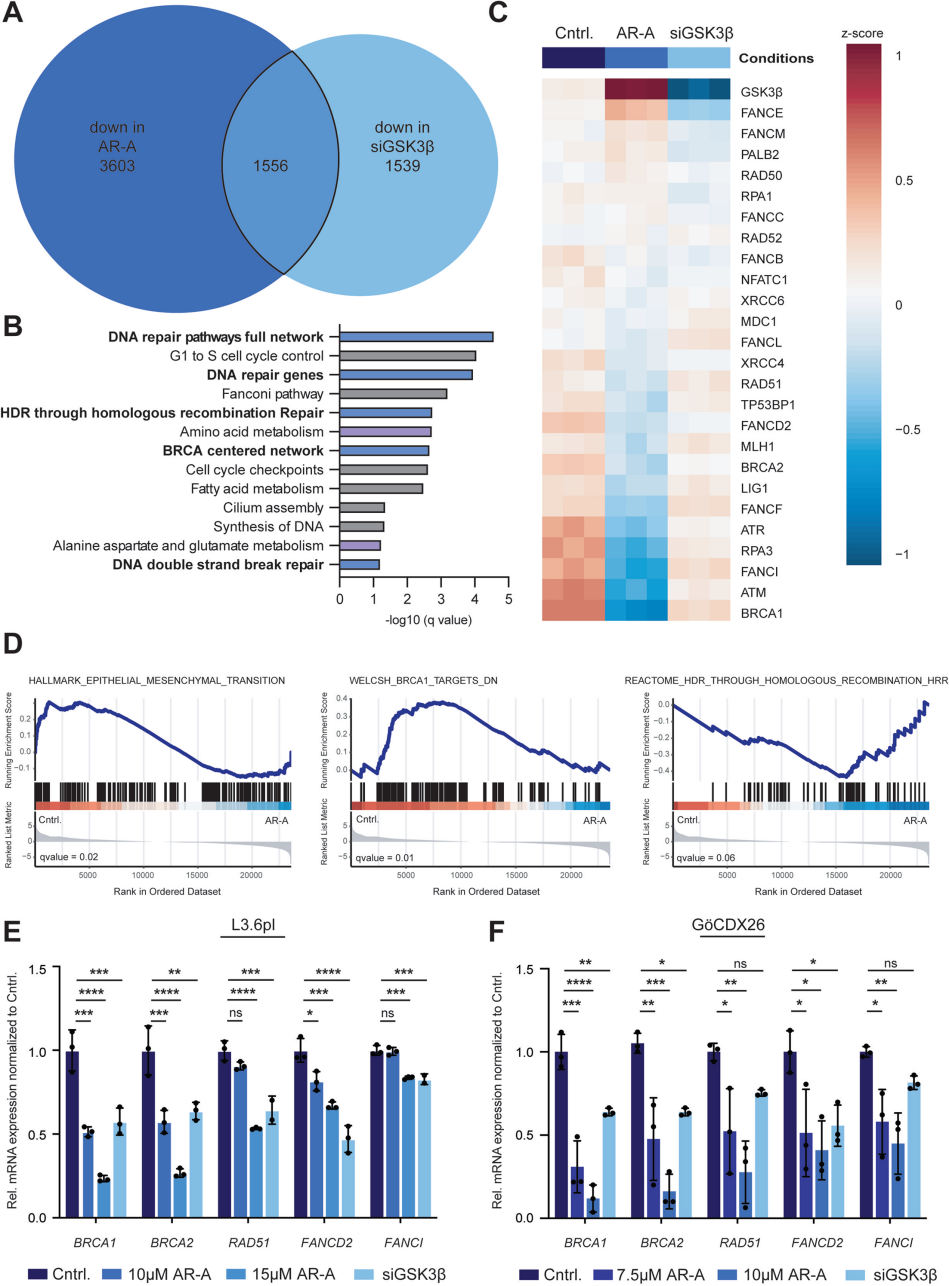
GSK3β regulates HR repair gene signatures. (**A**) Venn diagram indicating number of downregulated genes following either AR-A treatment or siGSK3β. 1556 genes were jointly downregulated by AR-A and siGSK3β. (**B**) Overlapping genes were used for gene enrichment analysis (pathway analysis; reactome.org), showing that DNA damage and DNA replication pathways were among the most significantly regulated gene signatures. (**C)** Z-score-based heatmap displaying differential expression of target gene signatures involved in DNA damage repair. (**D**) GSEA of RNA-seq data from AR-A treated L3.6pl cells. GSEA analysis revealed treatment-induced genes related to EMT and HR. (**E–F**) Real-time polymerase chain reaction (RT-qPCR) validation of target genes, that is, BRCA1, BRCA2, RAD51, FANCD2 and FANCI in (**E**) L3.6pl cells and (**F**) GöCDX26 cells showing reduced expression upon treatment with increasing concentration of AR-A (GSK3β-i) or introducing siGSK3β. Data are shown as mean±SD. Statistical analysis was performed using one-way ANOVA with Dunnett’s post-test where *p≤0.05, **p≤0.01, ***p≤0.001, ****p≤0.0001. ANOVA, analysis of variance; GSEA, Gene Set Enrichment Analysis; GSK3β, glycogen synthase kinase-3β; GSK3β-i, GSK3β inhibitors; HR, homologous recombination; RNA-seq, RNA sequencing; RT-qPCR, real-time polymerase chain reaction; siGSK3β, silencing of GSK3β.

Next, we explored the mechanistic basis of GSK3β-regulated growth and performed transcriptome analysis in human and murine PDAC cells with high levels of the kinase. For this purpose, we extracted messenger RNA (mRNA) from L3.6pl cells following pharmacological inhibition (by AR-A) or genetic silencing (siGSK3β) of the kinase and carried out mRNA sequencing (mRNA-seq) analysis. Principal component analysis confirmed robust clustering by treatment groups (online supplemental figure S2A) and comparative gene expression analysis revealed an overlap of 1556 differentially downregulated genes following GSK3β inactivation (figure 2A). Reactome pathway and Gene Set Enrichment Analysis on mutually downregulated genes showed multiple gene signatures involved in DNA damage response (DDR) and HR repair (figure 2B–D). Noteworthy, similar results were obtained from additional mRNA-seq analyses performed in murine KPCbl6 cells generated from *LSL-Kras*^G12D/+^,*Trp53*^R172H/+^,*Pdx1-Cre* mouse tumours (online supplemental figure S2B and C). Real-time polymerase chain reaction (RT-qPCR) and western blot analysis confirmed the role of GSK3β in the transcriptional regulation of HR genes and DNA repair. Accordingly, inhibition of the kinase dose-dependently reduced the expression of BRCA1, BRCA2, RAD51 and members of the FANC gene family (eg, FANCD2 and FANCI) in human L3.6pl and patient-derived PDAC cells (eg, GöCDX26 and GöCDX13) and in murine KPCbl6 cells both on mRNA and protein levels (figure 2E and F; online supplemental figure S2D-G). Similar effects were observed following genetic depletion of GSK3β (siGSK3β) (figure 2E and F; online supplemental figure S2H). Together, these experiments showed that the nuclear GSK3β signalling promotes the transcription of core HR genes, and in addition, suggests that this function is likely involved in the regulation of PDAC growth and DNA damage repair.

### GSK3β^high^/NFATc1^high^ subtype identification and implication in HR gene regulation

**Figure 3:**
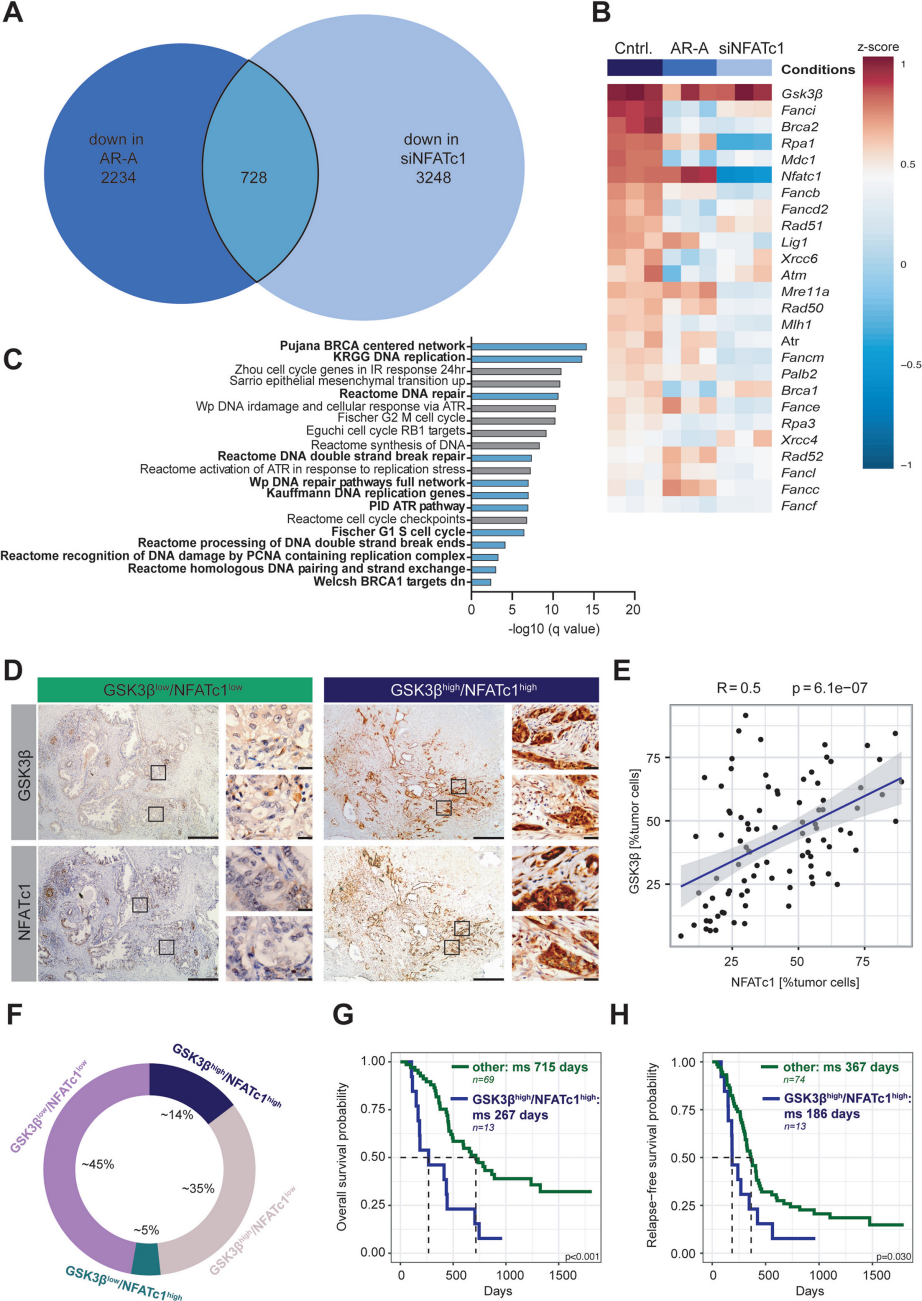
Nuclear GSK3β-NFATc1 promotes HR repair and tumour growth. (**A**) Overlap of downregulated gene signatures under AR-A treatment and siNFATc1 in KPCbl6 cells. (**B**) Z-scor-based heatmap displaying differential expression of target gene signatures involved in DNA damage repair following AR-A and siNFATc1 treatment for 48 hours. (**C**) Pathway enrichment analysis (GO analysis), showing the top enriched pathways related to DNA damage repair and replication. (**D**) Representative IHC images of GSK3β and NFATc1 in resected PDAC specimens. Scale bar indicates 500 µm and 100 µm, respectively. (**E**) Dot plot displaying correlation of nuclear GSK3β and NFATc1 expression in PDAC tumour samples. (**F**) Cut-off-based subtyping of PDAC tumours into GSK3β (high/low) and NFATc1 (high/low) subgroups. (**G–H**) Kaplan-Meier plots displaying (**G**) overall and (**H**) relapse-free survival of patients with GSK3β^high^/NFATc1^high^ subtype PDAC versus others. GO, gene ontology; GSK3β, glycogen synthase kinase-3β; HR, homologous recombination; IHC, immunohistochemistry; PDAC, pancreatic ductal adenocarcinoma.

Our objective was to identify the underlying transcriptional mechanisms in GSK3β-regulated DNA repair. GSK3β modulates various transcription factors and chromatin-associated proteins through site-specific phosphorylation.^33 34^ Among these transcription factors is the Ca^2+^-responsive transcription factor NFATc1,^21 34^ which exhibits oncogenic properties in PDAC. Previously, we demonstrated that nuclear GSK3β interacts with and safeguards NFAT proteins from rapid inactivation and degradation in fast growing tumour cells,^23 35^ and consistently, kinase inhibition dose-dependently reduced expression of NFATc1 in PDAC cells with high levels of GSK3β (online supplemental figure S3A and B). Subsequently, we investigated the role of NFATc1 in GSK3β-mediated transcriptional regulation of DDR and HR repair mechanisms involved in PDAC growth and resistance. To this end, we conducted mRNA-seq following GSK3β inhibition and genetic silencing of NFATc1 in KPCbl6 cells and identified a significant overlap of GSK3β and NFATc1 downregulated gene signatures (figure 3A). Importantly, the concurrent loss of GSK3β and NFATc1 led to the downregulation of 728 genes, all of which are linked to replication stress, DDR (eg, ATR/CHK1 checkpoint signalling), and HR-mediated DNA repair mechanisms, such as BRCA1, BRCA2 and RAD51 (figure 3B and C). These results underscore the importance of GSK3β and NFATc1 in the transcriptional regulation of key DDR and HR repair genes, which in turn are crucial for resistance and disease recurrence in PDAC. We then assessed the clinical relevance of these findings and determined the expression of the nuclear GSK3β-NFATc1 axis in resected tumours from the KFO5002 cohort. The workflow of these studies is depicted in online supplemental figure S3C. Importantly, IHC staining and subsequent QuPath-based analysis led to the identification of a subgroup of resected PDAC tissues characterised by a high percentage of co-expressing nuclear GSK3β and NFATc1, which we will refer to as GSK3β^high^/NFATc1^high^ PDAC subtype tumours (figure 3D and E). The GSK3β^high^/NFATc1^high^ subtype represents approximately 14% of resected PDAC cases and is associated with a particularly aggressive phenotype, predicting a significant reduction in both overall and relapse-free survival (figure 3F–H; online supplemental figure S3D-I). Patients with GSK3β^high^/NFATc1^high^ tumours experienced a significantly shorter relapse-free survival (186 vs 367 days; p=0.030) and overall survival (267 vs 715 days; p<0.001) compared with the remaining patient cohort (figure 3G and H). Further analyses revealed no significant correlation between GSK3β^high^/NFATc1^high^ tumours and histological grading, tumour stage (pT) (online supplemental figure S3J and K), or the presence of classical and basal-like features (data not shown). Collectively, these findings highlight the existence of a distinct PDAC subtype characterised by the activation of nuclear GSK3β-NFATc1 signalling and presumably involved in the transcriptional regulation of HR-mediated DNA repair mechanisms. Furthermore, GSK3β^high^/NFATc1^high^ tumours indicate an aggressive subtype with a high recurrence rate and an unfavourable prognosis following resection.

### Nuclear GSK3β-NFATc1 signalling promotes cisplatin-induced DNA damage repair

**Figure 4:**
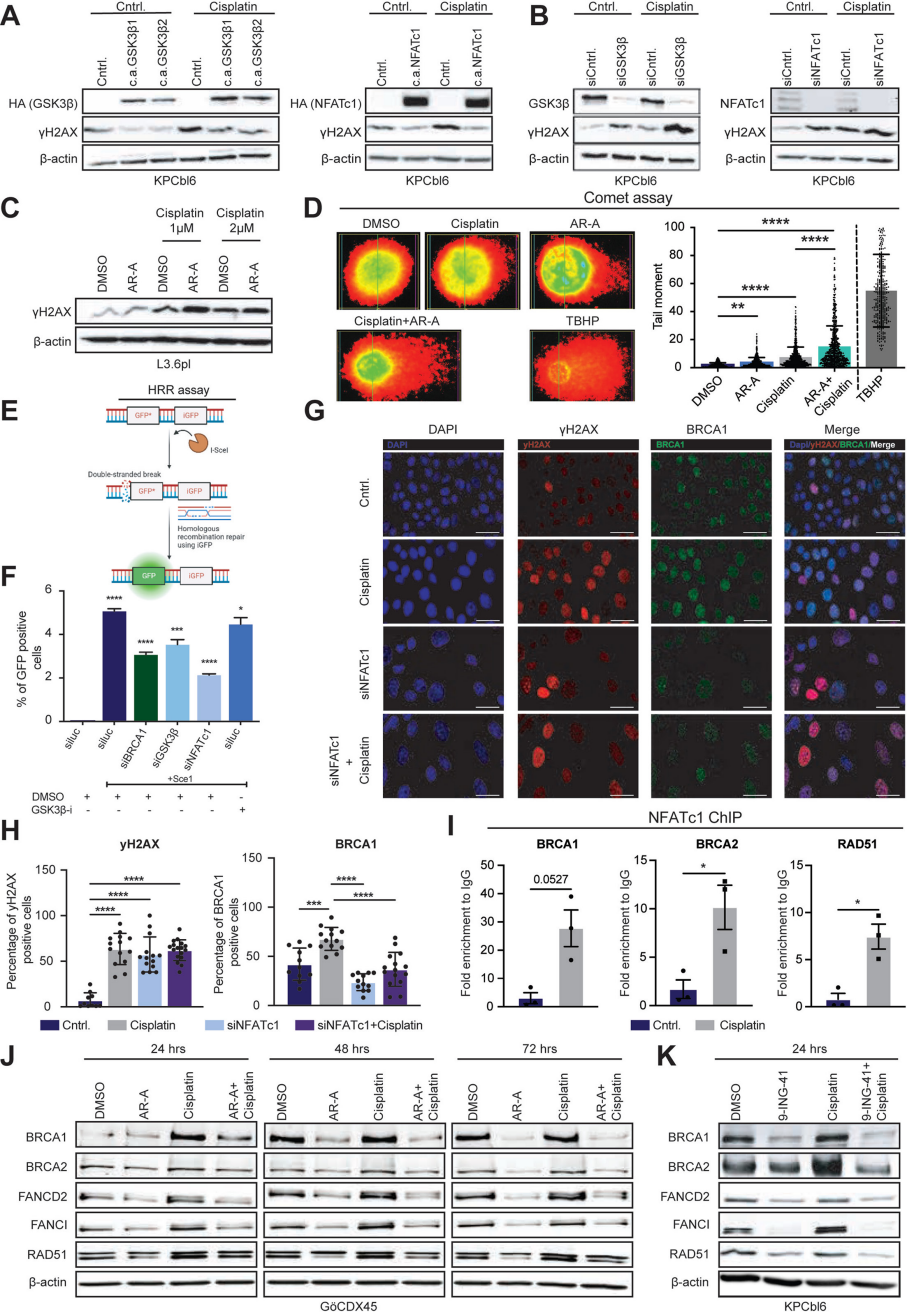
Inactivation of GSK3β-NFATc1 signalling impairs HR repair. (**A**) DNA damage was assessed by γH2AX protein levels in KPCbl6 cells following overexpression of HA-tagged GSK3β and NFATc1, respectively, alone or in combination with 1 µM cisplatin for 24 hours. (**B**) γH2AX protein levels in KPCbl6 cells following siRNA mediated knockdown of GSK3β and NFATc1, respectively, alone or in combination with 1 µM cisplatin for 48 hours. (**C**) Western blot displaying γH2AX protein expression in L3.6pl cells after 24 hours exposure to AR-A alone or in combination with increasing concentration of cisplatin (1 µM and 2 µM). (**D**) Representative images of alkaline comet assay and quantitative analysis of mean tail moment in KPCbl6 cells treated with 10 µM AR-A, 1 µM cisplatin or a combination of both drugs for 24 hours. 300 µM TBHP treatment for 2 hours was used as positive control for DNA strand breaks. (**E**) Schematic illustration of HR repair assay. (**F**) Homologous recombination was assessed in DRGFP expressing HCT116 cells transfected with empty vector or plasmid expressing SceI following siRNA mediated silencing of BRCA1, NFATc1 and GSK3β. (**G**) Representative images of BRCA1 and γH2AX IF in KCPbl6 cells with NFATc1 silencing alone or combined with cisplatin (1 µM) for 24 hours. (**H**) Quantitative analysis of BRCA1 and γH2AX in KCPbl6 cells with NFATc1 silencing alone and in combination with cisplatin (1 µM). (**I**) ChIP-assay displaying NFATc1 DNA occupancy at BRCA1, BRCA2 and RAD51 regulatory binding elements following 1 µM cisplatin treatment in KNPC cells for 24 hours. (**J**) Immunoblot showing time-dependent loss of HR gene signatures in GöCDX45 cells following treatment with 10 µM AR-A, 1 µM cisplatin or combination of both drugs for 24, 48 and 72 hours, respectively. (**K**) Protein expression of HR genes in KPCbl6 cells following 24 hours exposure to 1 µM 9-ING-41 and 1 µM cisplatin alone and in combination. Data are shown as mean±SD. Statistical analysis was performed using one-way ANOVA with Dunnett’s post-test, where *p≤0.05, **p≤0.01, ***p≤0.001, ****p≤0.0001. ANOVA, analysis of variance; ChIP, chromatin immunoprecipitation; GSK3β, glycogen synthase kinase-3β; HR, homologous recombination.

We then examined the role of nuclear GSK3β-NFATc1 signalling in chemotherapy-induced DNA damage and repair. Currently, platinum-based regimens such as FOLFIRINOX and NALIRIFOX are the most widely used treatments in both adjuvant therapy and palliative care of metastatic PDAC.^3 7 36^ Mechanistically, platinum compounds (eg, cisplatin, oxaliplatin) induce DNA damage through various pathways including the formation of interstrand crosslinks,^37–39^ which in turn can progress to severe DNA DSBs. DSBs activate specific DDR mechanisms, such as ATR/TopBP1/CHK1 checkpoint pathway and subsequent initiation of HR mediated DNA repair.^27 40–43^ The following results support a prominent role of nuclear GSK3β-NFATc1 signalling in platinum-induced DDR and DNA repair processes: first, cisplatin treatment of GSK3β^high^/NFATc1^high^ cells induced ATR/TopBP1/CHK1 checkpoint signalling, as indicated by CHK1 phosphorylation at Ser-345 (online supplemental figure S4A). In contrast, the application of GSK3β-i (AR-A and 9-ING-41) antagonised basal and cisplatin-induced checkpoint activation, thus leading to a reduction in ATR/TopBP1 expression and prevention of CHK1 phosphorylation (online supplemental figure S4A). Second, the introduction of either constitutively active GSK3β (c.a.GSK3β) or NFATc1 (c.a.NFATc1) accelerated the repair of both endogenous and treatment-induced DSBs in PDAC, as evidenced by a significant reduction in γH2AX phosphorylation levels (figure 4A). Third, inactivation of the pathway, either through genetic depletion of GSK3β (siGSK3β) and NFATc1 (siNFATc1), or treatment with specific GSK3β-i, resulted in accumulation of DSBs in KPCbl6 and L3.6pl PDAC cells (figure 4B and C). Consistently, exacerbation of DSBs was reflected by increased γH2AX phosphorylation and accelerated nucleotide tail moment in alkaline comet assays (figure 4D; online supplemental figure S4B-D). We next examined whether nuclear GSK3β-NFATc1 signalling promotes DSB repair through activation of HR. To this end, we employed cancer cells with a chromosomally integrated HR reporter gene containing an I-SceI recognition sequence (figure 4E). In these cells, successful DNA damage repair by the HR pathway is indicated by induced GFP expression, which can be quantified by flow cytometry (figure 4F). Importantly, silencing of NFATc1, and to a lesser extent inhibiting GSK3β, strongly impaired HR-mediated repair (figure 4F), and this effect was associated with accelerated DSBs and a significant reduction of damage-related foci formation in core HR repair genes, such as BRCA1 and RAD51, in GSK3β^high^/NFATc1^high^ cells (figure 4G and H; online supplemental figure S4E and F). Finally, chromatin immunoprecipitation experiments and expression analyses revealed cisplatin-induced NFATc1 binding at BRCA1, BRCA2 and RAD51 regulatory gene sequences (figure 4I) and confirmed successful prevention of drug-induced HR gene expression when co-treated with GSK3β-i (figure 4J and K). Consistently, similar suppression of HR gene expression was observed in KPCbl6 cells on co-treatment of oxaliplatin with the GSK3β inhibitor 9-ING-41 (online supplemental figure S4G).

### NFATc1 activation is required for cisplatin-induced resistance of GSK3β^high^/NFATc1^high^ PDAC

**Figure 5:**
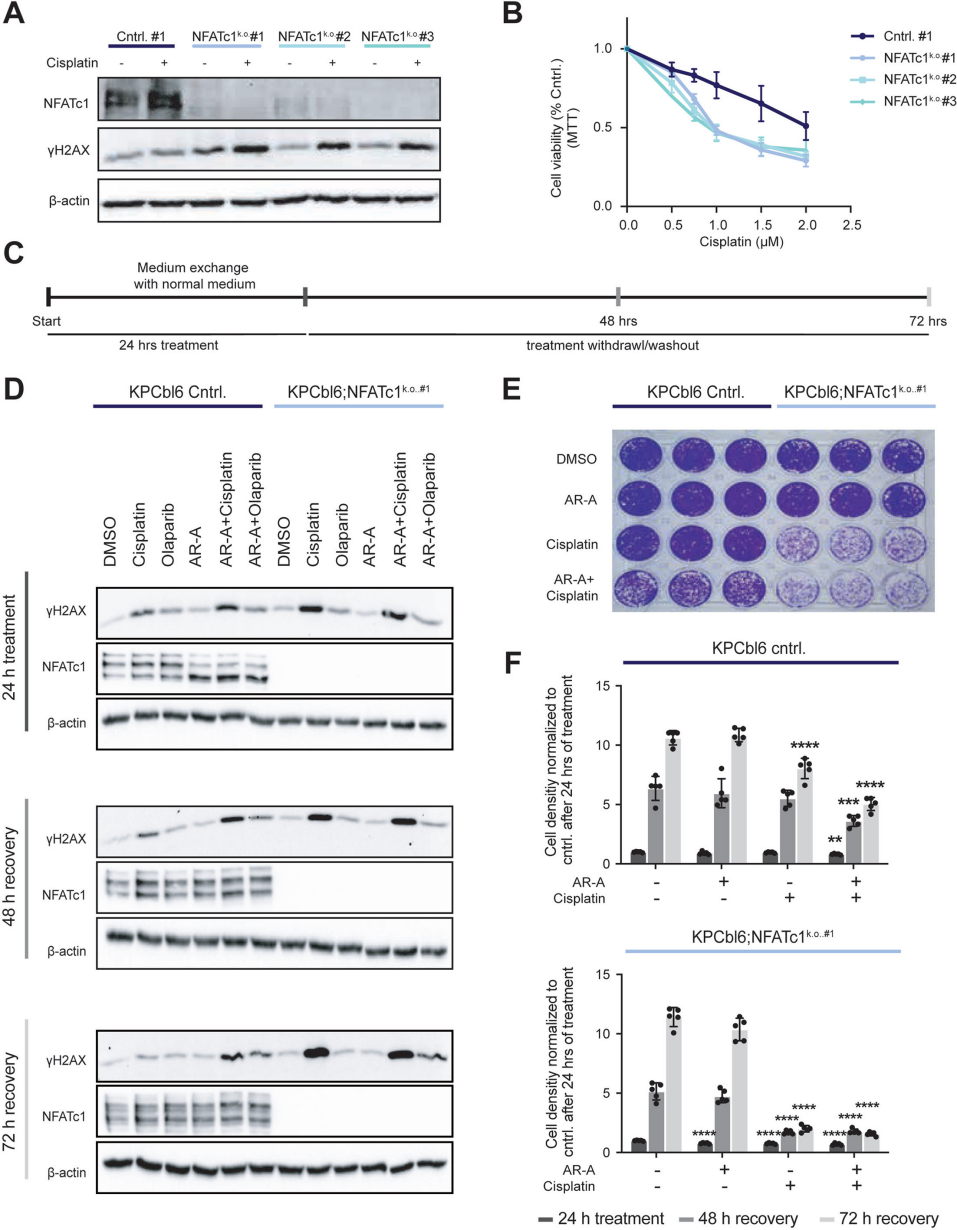
NFATc1 activation is required for DNA damage repair and recovery. (**A**) Protein expression of NFATc1 and γH2AX in KPCbl6 cntrl. and CRISPR KPCbl6;NFATc1^k.o^. clones following treatment with 1 µM cisplatin for 24 hours. (**B**) MTT assay displaying decreased viability of KPCbl6;NFATc1^k.o^. cell clones when treated with increasing concentrations of cisplatin for 72 hours. (**C**) Schematic workflow of cell recovery experiment. (**D**) DNA damage repair in KPCbl6 cntrl. and KPCbl6;NFATc1^k.o^ cells following single or combined treatment with 1 µM cisplatin, 10 µM olaparib and 10 µM AR-A for 24 hours. On treatment termination, cells were cultured in drug-free media for an additional 48 and 72 hours. Time-dependent DNA damage resolution was determined by γH2AX expression. Note that AR-A treatment and NFATc1 depletion both prevented cells from successful DSBs repair, even up to 72 hours on cisplatin withdrawn. PARP-inhibition by olaparib was used as internal control. (**E**) Representative images of crystal violet staining in KPCbl6 cntrl. and KPCbl6;NFATc1^k.o^. cells following recovery from cisplatin treatment for 72 hours. (**F**) Quantitative analysis of cell density in KPCbl6 cntrl and KPCbl6;NFATc1^k.o^. cells. Cells were treated with cisplatin for 24 hours and subsequently cultured in fresh media for an additional 48 and 72 hours. Data are shown as mean±SD; statistical analysis was performed using one-way ANOVA with Dunnett’s post-test with ***p≤0.001 and ****p≤0.0001. ANOVA, analysis of variance; DSB, double-strand break; MTT, 3-[4,5-dimethylthiazol-2-yl]-2,5 diphenyl tetrazolium bromide.

The results thus far indicated a pivotal role of the GSK3β-NFATc1 pathway in the transcriptional regulation of HR-mediated DSBs repair. Subsequent studies examined whether NFATc1-induced DNA damage repair indeed contributes to platinum resistance and, if so, whether inactivation of the transcription factor increases drug efficacy in GSK3β^high^/NFATc1^high^ PDAC. For this purpose, we employed murine KNPC cells with constitutive nuclear activation of NFATc1 (generated from genetically modified *LSL-Kras*^G12D/+^, NFATc1^c^.^a^.*,Trp53*,*p48-Cre* mice)^44^ and KPCbl6 cells with CRISPR/Cas9-mediated NFATc1 deletion (referred to as KPCbl6;NFATc1^k.o^ cells). Collectively, the growth and resistance studies revealed the following key findings. KNPC cells with nuclear NFATc1 activation displayed significantly reduced sensitivity to cisplatin-induced growth retardation when compared with KPCbl6 control cells (online supplemental figure S5A). By contrast, genetic depletion of NFATc1 slightly increased basal DSBs (figure 5A, online supplemental figure S5B) but rendered KPCbl6;NFATc1^k.o^ cells far more sensitive to cisplatin treatment, reflected by a further increase in γH2AX (figure 5A) and a tremendous reduction of tumour cell viability (figure 5B) and proliferation (online supplemental figure S5C). The loss of NFATc1 persistently prevented DNA damage repair and regeneration of PDAC tumour cell growth even 72 hours after cisplatin removal (figure 5C–F). However, similar effects on γH2AX phosphorylation, cell viability and proliferation were observed in KPCbl6 cells following combined treatment with cisplatin and GSK3β-i but not in KPCbl6;NFATc1^k.o^ cells during either the treatment or recovery phase (figure 5D–F; online supplemental figure S5C and D).

In summary, these findings demonstrate the critical role of nuclear NFATc1 in GSK3β-driven DSBs repair and resistance to cisplatin. In addition, our results also suggest that targeted disruption of the pathway would offer novel therapeutic strategies to prevent cisplatin resistance in patients with GSK3β^high^/NFATc1^high^ subtype tumours.

### GSK3β targeting requires HR proficiency to exacerbate platinum-mediated growth suppression

**Figure 6:**
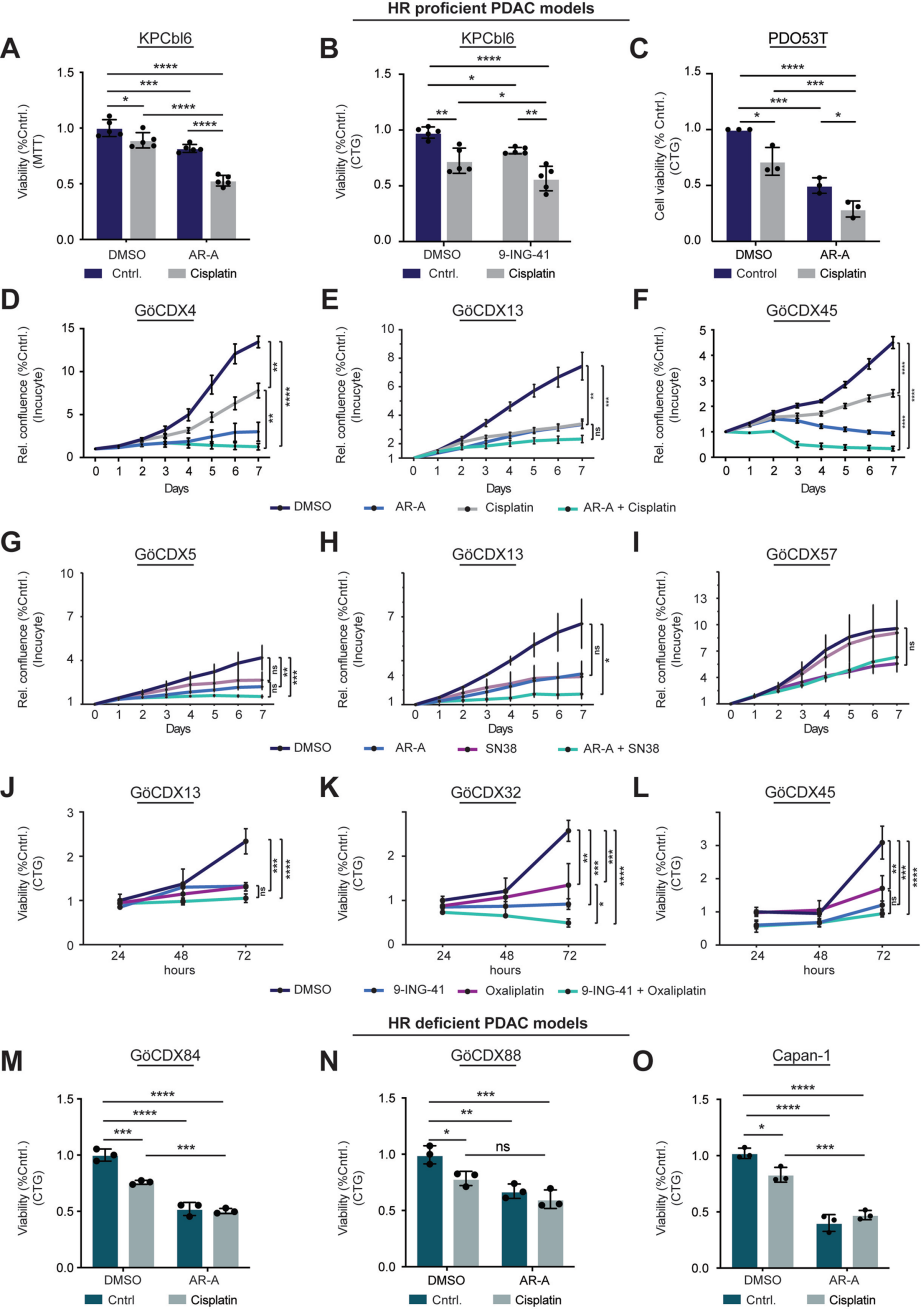
Inhibition of nuclear GSK3β-NFATc1 signalling sensitises HR-proficient PDAC cells to platinum-based chemotherapy. (**A**) Cell viability was determined by MTT assays in KPCbl6 cells after treatment with 10 µM AR-A, 1 µM cisplatin and combination of both drugs for 48 hours. (**B**) CTG assays were conducted in KPCbl6 cells after treatment with 1 µM 9-ING-41, 1 µM cisplatin or combination for 48 hours. (**C**) CTG assay performed in PDAC organoids (PDO53T) following treatment with 6 µM cisplatin, 15 µM AR-A or both drugs for 72 hours. Data are shown as mean±SD. (**D–F**) IncuCyte was carried out to determine time-dependent cell proliferation in HR-proficient (**D**) GöCDX4, (**E**) GöCDX13 and (**F**) GöCDX45 cells following exposure to 5 µM AR-A, 1 µM cisplatin and a combination of both drugs. Statistical analysis of cell proliferation was performed at day 7. Data are shown as mean±SEM. (**G–I**) Tumour cell proliferation was determined by IncuCyte in (**G**) GöCDX5, (**H**) GöCDX13 and (**I**) GöCDX57 cells following treatment with 5 µM AR-A, 5 µM SN38 or combination of both drugs up to 7 days. Statistical analysis of cell proliferation was performed at day 7. Data are shown as mean±SEM. (**J–L**) Tumour cell viability was determined by CTG in (**J**) GöCDX13, (**K**) GöCDX32 and (**L**) GöCDX45 cells following treatment with 250 nM 9-ING-41 and 1 µM oxaliplatin alone or in combination up to 72 hours. Statistical analysis of cell proliferation was performed at 72 hours. (**M–O**) Drug-induced effects on cell viability were measured by CTG in BRCA2 mutated (**M**) GöCDX84, (**N**) GöCDX88 and (**O**) Capan-1 PDAC cells following treatment with 10 µM AR-A, 2 µM cisplatin or drug combination. Data are shown as mean±SD. Statistical analysis was performed using one-way ANOVA with Dunnett’s post-test where *p≤0.05, **p≤0.01, ***p≤0.001, ****p≤0.0001. ANOVA, analysis of variance; CTG, CellTiter-Glo; HR, homologous recombination; MTT, 3-[4,5-dimethylthiazol-2-yl]-2,5 diphenyl tetrazolium bromide; PDAC, pancreatic ductal adenocarcinoma.

We next examined the relevance of HR proficiency for the efficacy of combined treatment with cisplatin and GSK3β-i. To this end, we used GSK3β^high^/NFATc1^high^ PDAC cell lines and PDOs, both with and without genetic alterations of BRCA2, the most prevalent mutation in HR repair genes. The administration of cisplatin or oxaliplatin alone resulted in a moderate reduction of cell viability and growth across various cell lines (KPCbl6, GöCDX4, GöCDX13 and GöCDX45) and PDOs (eg, PDO53T). This was validated through CellTiter-Glo assays and IncuCyte Live Cell imaging analysis (figure 6A–F; online supplemental figure S6A and B). Notably, the co-treatment with GSK3β-i significantly accelerated cisplatin-induced growth retardation over time in all tested GSK3β^high^/NFATc1^high^ PDAC models with proficient HR repair (figure 6A–F). Likewise, GSK3β-i treatment also increased the efficacy of other HR-promoting agents, particularly irinotecan (SN38; figure 6G–I) and oxaliplatin (figure 6J–L). However, GSK3β-i failed to sensitise PDAC cell lines (GöCDX84, GöCDX88 and Capan-1) with HR deficiency due to mutations of BRCA2 (figure 6M–O).

These results support the prevailing concept that co-treatment with specific GSK3β-i can enhance the therapeutic potential of DSB-inducing agents in the treatment of GSK3β^high^/NFATc1^high^ PDAC, particularly in tumours with proficient HR repair capabilities.

### Targeting GSK3β-NFATc1 signalling prevents DNA repair and overcomes cisplatin resistance in preclinical PDAC models

**Figure 7:**
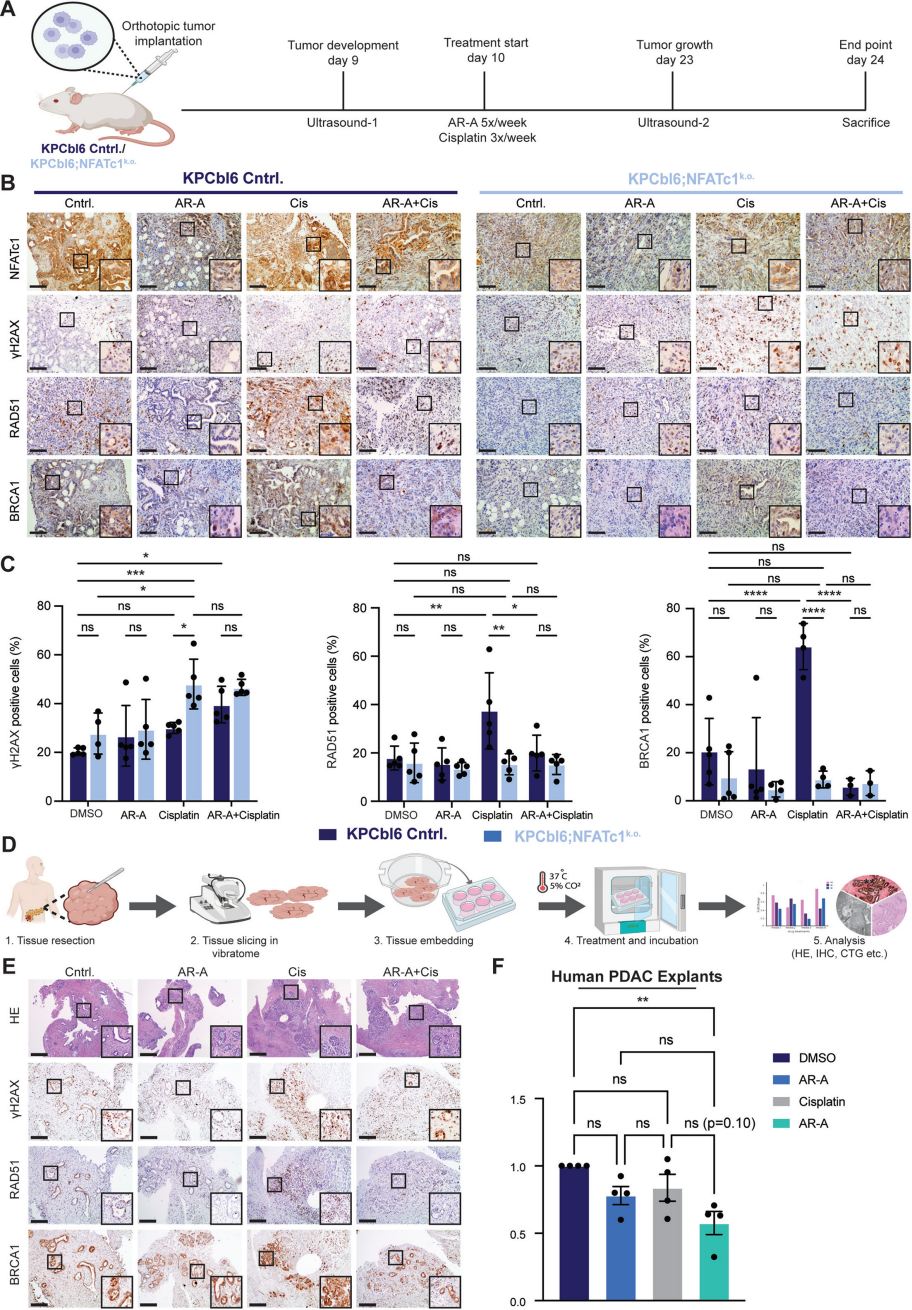
GSK3β-NFATc1 targeting prevents HR-induced repair and increases cisplatin efficacy in preclinical PDAC models. (**A**) Schematic illustration displaying orthotopic mouse model and the respective treatment regimen. (**B**) Representative IHC images of NFATc1, γH2AX, RAD51 and BRCA1 in KPCbl6 cntrl. and KPCbl6;NFATc1^k.o^. tumour tissues following treatment. Scale bar indicates 100 µm. (**C**) Quantitative analysis of γH2AX, RAD51 and BRCA1 IHC staining in cisplatin treated KPCbl6 cntrl. tumours when combined with GSK3β-i and following genetic depletion of NFATc1 (KPCbl6;NFATc1^k.o^ tumour). (**D**) Schematic illustration of human PDAC tissue slice preparation, subsequent treatment and analysis of ex vivo PDAC explants. (**E**) Representative H&E images and IHC staining for γH2AX, RAD51 and BRCA1 expression in human PDAC explants on treatment. Scale bar indicates 200 µm. (**F**) Cell viability was determined in human PDAC explants by CTG assays after 48 hours treatment with 10 µM AR-A, 2.5 µM cisplatin or a combination of both drugs, n=4. Data are shown as mean±SD. Statistical analysis was performed using two-way ANOVA (**C**) and one-way ANOVA (**F**) with Dunnett’s post-test, where *p≤0.05, **p≤0.01, ***p≤0.001, ****p≤0.0001. ANOVA, analysis of variance; CTG, CellTiter-Glo; GSK3β-i glycogen synthase kinase-3β inhibitors; HR, homologous recombination; IHC, immunohistochemistry; PDAC, pancreatic ductal adenocarcinoma.

Finally, we examined the therapeutic potential of combination treatment and took advantage of a syngeneic PDAC tumour mouse model and ex vivo human PDAC explants. For the syngeneic tumour model, we injected KPCbl6 control and KPCbl6;NFATc1^k.o^ cells into the pancreas of immunocompetent C57BL/6 mice. Tumour growth was monitored by ultrasound before (day 9) and at the end of treatment (figure 7A). Therapy was initiated on day 10 by intraperitoneal administration of either cisplatin (4 mg/kg 3 ×/week), AR-A (10 mg/kg 5 ×/week) or a combination of both drugs for 2 weeks. Subsequently, we sacrificed mice for histological analysis (figure 7A–C). The treatment of mice with cisplatin resulted in moderate growth suppression of GSK3β^high^/NFATc1^high^ KPCbl6 tumours (online supplemental figure S6A). However, sensitivity to cisplatin was significantly higher in KPCbl6;NFATc1^k.o^ tumours or following application of GSK3β-i (online supplemental figure S6A), and these effects were accompanied by reduced HR gene expression (eg, BRAC1 and RAD51) and significant accumulation of DSBs (figure 7B and C). Most importantly, co-treatment with GSK3β-i did not enhance the efficacy of cisplatin in NFATc1-deficient tumours and therefore, no significant changes in growth retardation or γH2AX accumulation were observed in KPCbl6;NFATc1^k.o^ tumours (figure 7B–C, online supplemental figure S6C). These findings emphasised the exceptional role of NFATc1 in nuclear GSK3β-mediated DNA repair and, in addition, supported the idea that GSK3β primarily signals via NFATc1 to promote resistance against cisplatin.

Lastly, we carried out precision cut slices from human PDAC specimens of the KFO5002 cohort and generated ex vivo tumour explants for subsequent preclinical drug validation (figure 7D). To this end, four surgically resected GSK3β^high^/NFATc1^high^ tumour tissues (online supplemental figure S6D) were sliced and cultured on gelatin sponges, as described previously.^45^ Figure 7D illustrates the stepwise preparation, treatment and consecutive analysis of PDAC explants. Consistent with previous reports, PDAC explants remained viable for at least 4–5 days and maintained their tumour architecture on preparation, making this model a valuable preclinical tool for drug treatment analysis. We started treatment of GSK3β^high^/NFATc1^high^ PDAC explants at day 1 and applied cisplatin (2.5 μΜ), AR-A (10 μΜ) or a combination of both drugs for 48 hours. Consistent with results from PDOs and syngeneic KPCbl6 mouse tumours, GSK3β^high^/NFATc1^high^ PDAC explants from all four patients were only marginally sensitive to single drug treatment (figure 7D–F). However, combined treatment with GSK3β-i and cisplatin led to a significant downregulation of HR genes and accumulation of DSBs and, in addition, resulted in significant reduction of tumour viability (figure 7E and F).

## Discussion

Growing evidence from recent preclinical and clinical studies supports the concept of subtype-specific targeting strategies in PDAC.^8–10 12–14^ This is especially relevant for PDAC with mutations in HR repair genes, such as BRCA1 and BRCA2, which often lead to increased sensitivity against platinum-based chemotherapy.^19–22^ Accordingly, current international guidelines recommend platinum-based therapies, such as FOLFIRINOX, as the first-line treatment for patients with PDAC with germline defects in BRCA genes.^19–21^ However, apart from the HR deficiency subtype, only a limited number of stratification-based tailored treatment approaches have entered clinical practice. Notable exceptions include the use of checkpoint inhibitors for treatment of PDAC with MSI^high^, larotrectinib for tumours with NTRK fusions, and the anticipated US Food and Drug Administration approval of mutant-specific KRAS^G12C^ and KRAS^G12D^ inhibitors for the treatment of metastatic PDAC.^10 14 15 46–50^

With the identification of GSK3β^high^/NFATc1^high^ tumours, we expand the number of PDAC subtypes with clinical relevance and potential for stratification-based treatment. This novel subtype is characterised by nuclear co-accumulation of the GSK3β kinase and the transcription factor NFATc1, accounts for approximately 14% of resected PDAC and predicts a particularly aggressive and deadly phenotype. In fact, GSK3β^high^/NFATc1^high^ tumours relapse significantly earlier after surgical resection, and most patients rapidly succumb to disease progression. Therefore, from the clinical point of view, stratification for the GSK3β^high^/NFATc1^high^ PDAC subtype at the time of diagnosis appears reasonable to identify patients at particularly high risk for rapid tumour recurrence and early death.

Previous work has shown that activation of GSK3β can exhibit oncogenic or tumour-suppressor activities, depending on the tumour entity, subcellular localisation of the kinase and the selection of downstream target genes.^23 24 27–29 51^ For example, activation of GSK3β has been shown to promote cell proliferation and disease progression in colorectal cancer, PDAC and certain leukaemia subgroups.^52–54^ In this study, we confirm the protumourigenic potential of GSK3β in PDAC and provide evidence that this function requires the nuclear co-expression and activation of NFATc1. Increased expression and activation levels of NFATc1 were reported in advanced and in association with a rapidly growing tumour phenotype.^23 55^ NFATc1 belongs to the Ca^2+^-responsive NFAT family of inflammatory transcription factors^56 57^ and is involved in the regulation of adaptive cell processes, such as metabolism, growth and differentiation. The subcellular distribution pattern of NFAT proteins is tightly controlled by a complex network of signalling-regulated kinases and phosphatases.^57^ For instance, phosphorylation by DYRK, casein kinase II or protein kinase A retains inactive NFAT proteins in the cytosol, while calcineurin-dependent dephosphorylation enables rapid nuclear translocation and activation of the factor.^57^ We have previously shown that nuclear GSK3β can target NFAT proteins for site-specific phosphorylation, thereby protecting the transcription factors from ubiquitination and subsequent proteasomal degradation in tumour cells.^23 35^ The identification of the GSK3β^high^/NFATc1^high^ subtype underscores the biological relevance of this signalling and transcription pathway in a subcohort of particularly aggressive PDAC, in which nuclear GSK3β appears to dominate other NFATc1-regulating kinases. Importantly, activation of the GSK3β-NFATc1 transcription pathway persists in preclinical human PDAC models, for example, PDX-derived tumour cells, organoids and ex vivo explants generated from human GSK3β^high^/NFATc1^high^ PDAC tumours and thus enabled us to perform in-depth mechanistic studies and functional analyses.

Mechanistically, nuclear GSK3β-NFATc1 signalling fosters PDAC cell growth and promotes cell viability and DNA repair through transcriptional regulation of genes involved in the regulation of cell cycle progression, replication stress and HR. Specifically, nuclear NFATc1 binds at and induces transcription from regulatory elements of BRCA1, BRCA2 and RAD51 genes for subsequent HR-mediated repair of DNA double-strand breaks. Of note, this mechanism is particularly evident in GSK3β^high^/NFATc1^high^ PDAC subtypes, where nuclear GSK3β-NFATc1 signalling drives resistance against platinum (eg, cisplatin and oxaliplatin) and irinotecan (SN38), which are both key components of FOLFIRINOX, the first-line regimen in adjuvant therapy and in the treatment of metastatic PDAC.

Thus, targeting nuclear GSK3β-NFATc1 signalling represents a promising strategy to inhibit HR-mediated DNA repair and thereby enhance the efficacy of platinum-based therapies in GSK3β^high^/NFATc1^high^ PDAC. Accordingly, disruption of nuclear GSK3β-NFATc1 signalling—either through genetic modification or following application of selective GSK3β inhibitors, for example, 9-ING-41—impairs DNA repair mechanisms and significantly increases the efficacy of cisplatin, as demonstrated in patient-derived PDAC cell lines, organoids and preclinical models of both murine and human GSK3β^high^/NFATc1^high^ PDAC.

Collectively, our study identifies a previously unrecognised GSK3β^high^/NFATc1^high^ PDAC subgroup that is characterised by a highly aggressive, rapidly relapsing and resistant disease phenotype. Furthermore, we dissected the underlying mechanisms in GSK3β^high^/NFATc1^high^ subtype progression and resistance and shed light on the complex processes through which the nuclear GSK3β-NFATc1 transcription pathway promotes HR gene regulation, DNA repair and resistance to platinum-based therapy. Consequently, our findings provide a molecular rationale and theoretical foundation for future clinical trials assessing the efficacy of combined treatment with GSK3β inhibitors and platinum-based chemotherapy in GSK3β^high^/NFATc1^high^ PDAC subtype tumours.

## Supplementary material

10.1136/gutjnl-2025-336227 online supplemental figure 1

10.1136/gutjnl-2025-336227 online supplemental figure 2

10.1136/gutjnl-2025-336227 online supplemental figure 3

10.1136/gutjnl-2025-336227 online supplemental figure 4

10.1136/gutjnl-2025-336227 online supplemental figure 5

10.1136/gutjnl-2025-336227 online supplemental figure 6

10.1136/gutjnl-2025-336227 online supplemental file 1

10.1136/gutjnl-2025-336227 online supplemental file 2

## Data Availability

Data are available upon reasonable request. All data relevant to the study are included in the article or uploaded as supplementary information.
