# Supplementary figures and images for: GSK3βhigh/NFATc1high subtype targeting overcomes therapy resistance in pancreatic cancer through transcriptional induction of homologous recombination repair
Source: Gut. 2025 Dec 31;75(8):e336227. doi: 10.1136/gutjnl-2025-336227 (PMC13422050; doi:10.1136/gutjnl-2025-336227)

**A**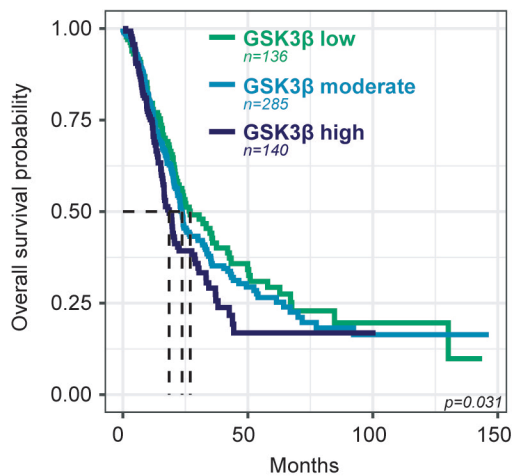**B**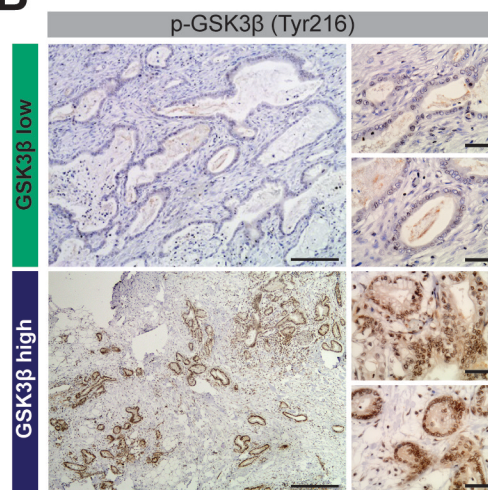**C**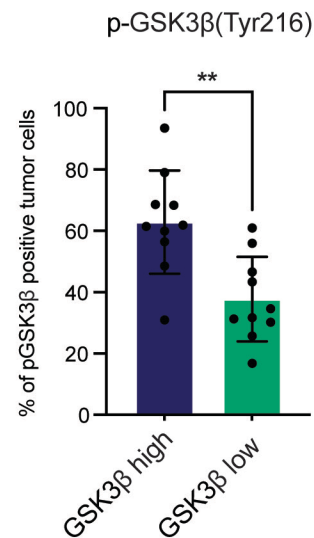**D**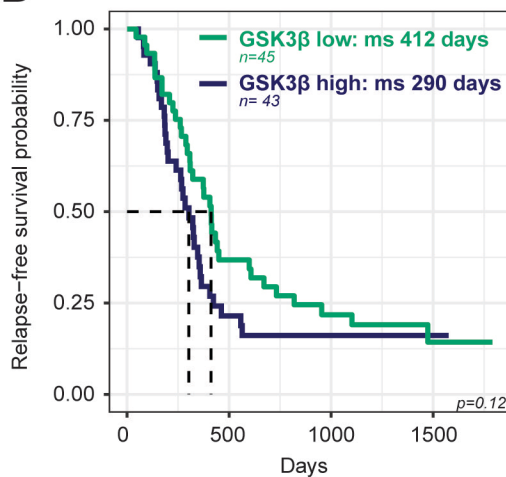**E**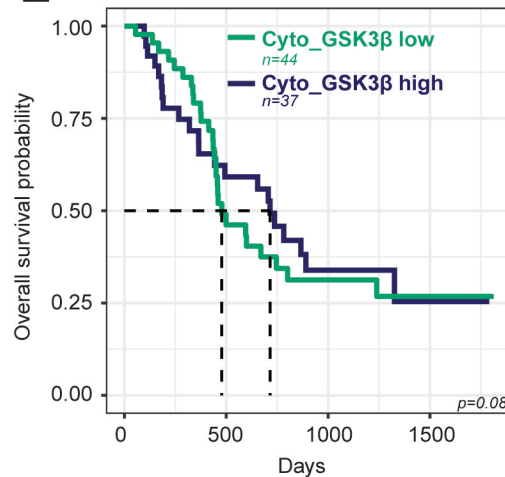**F**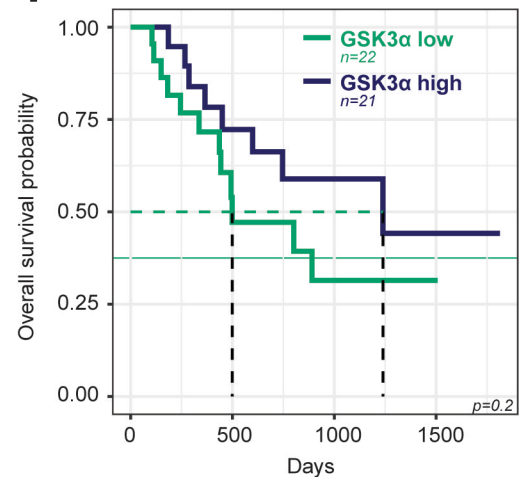**G**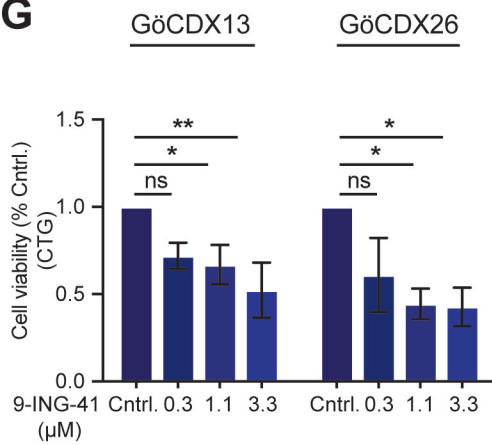**H**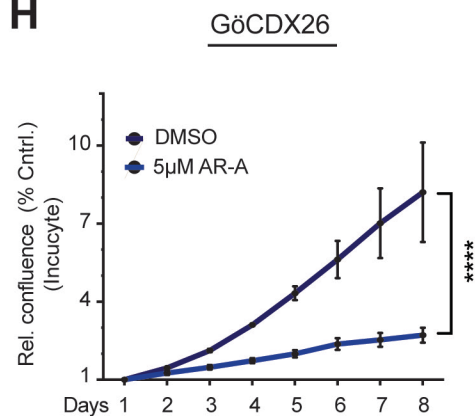**I**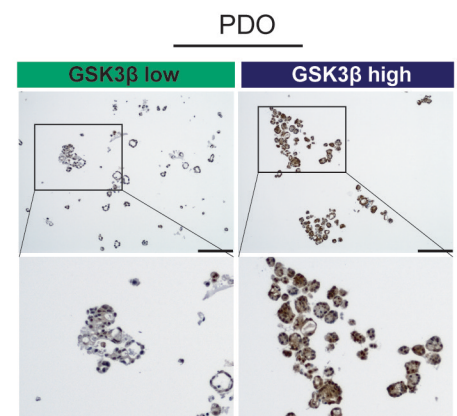

Supplement: online supplemental figure 1 [file gutjnl-75-8-s001.pdf]

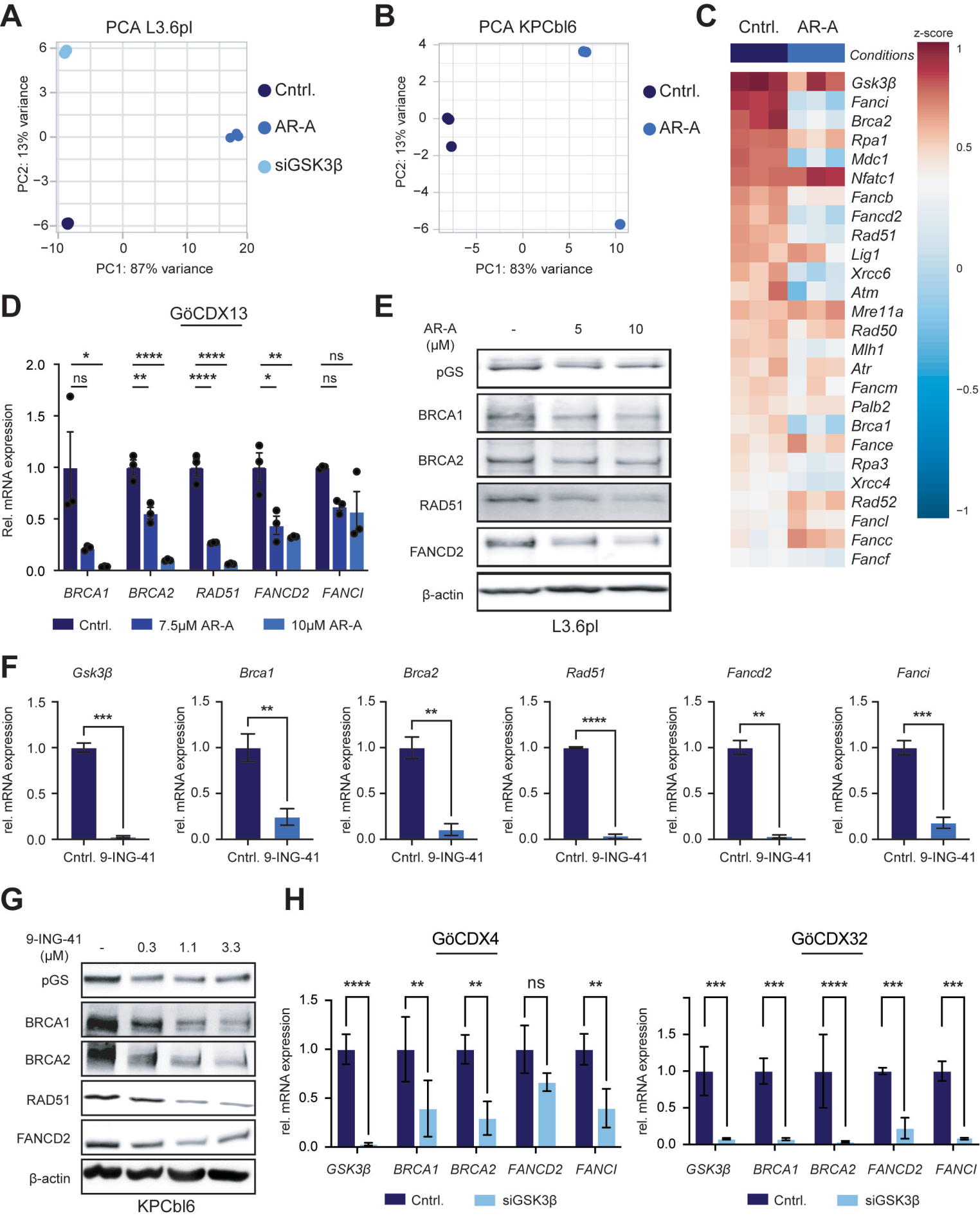

Supplement: online supplemental figure 2 [file gutjnl-75-8-s002.pdf]

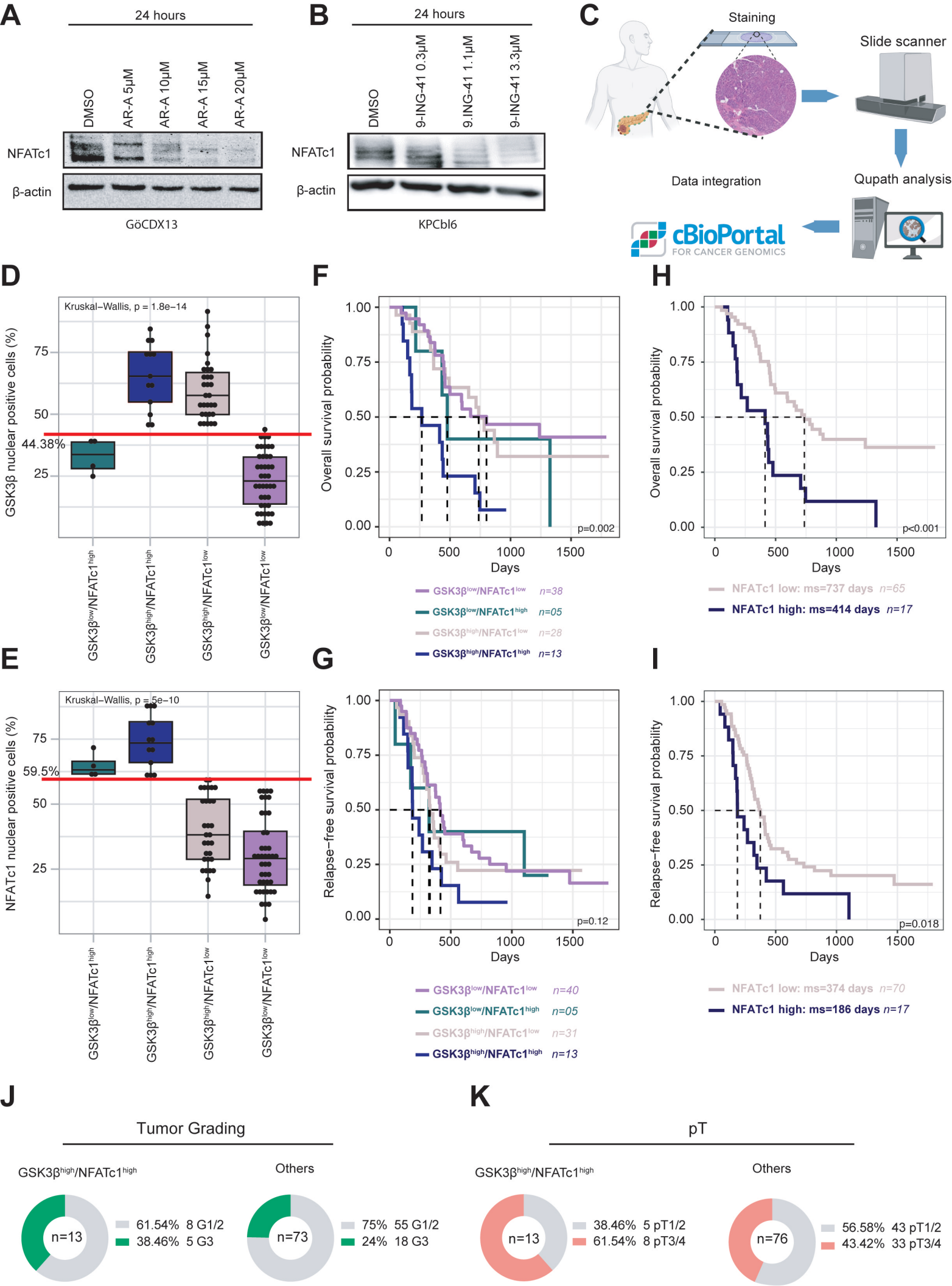

Supplement: online supplemental figure 3 [file gutjnl-75-8-s003.pdf]

**A**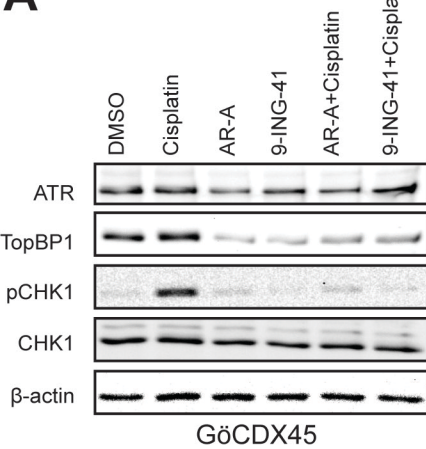**B**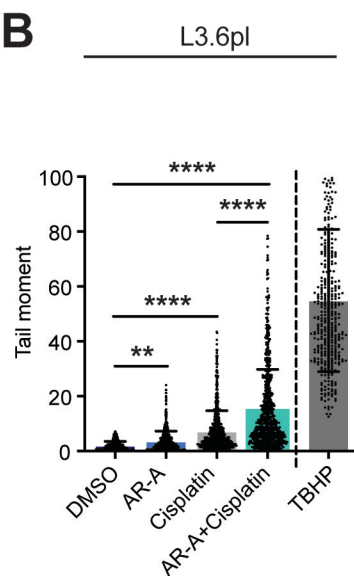**C**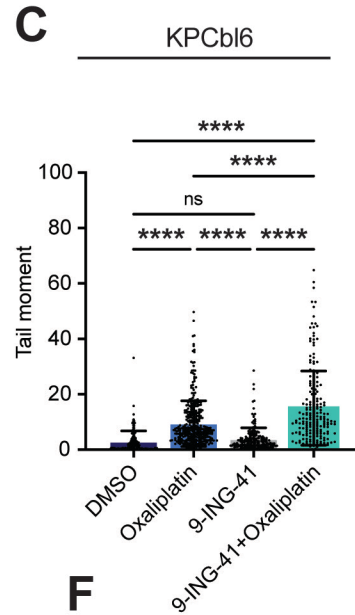**D**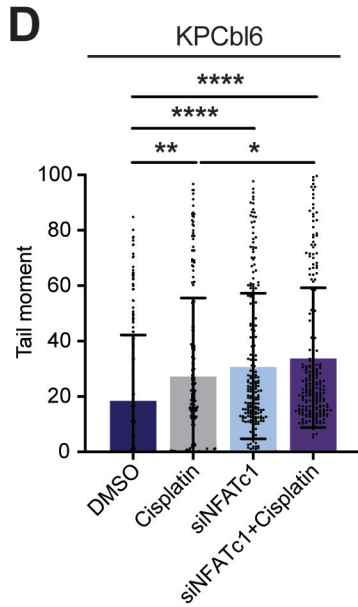**E**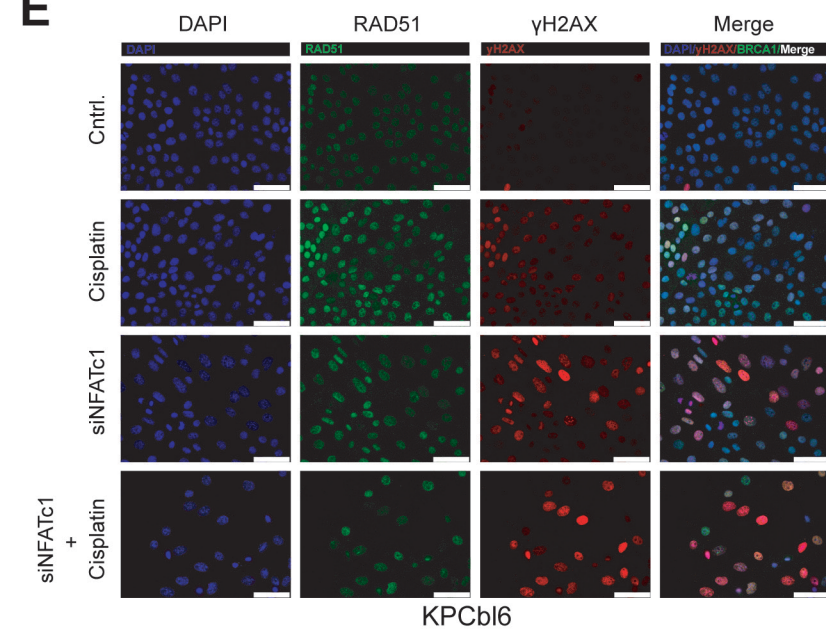**F**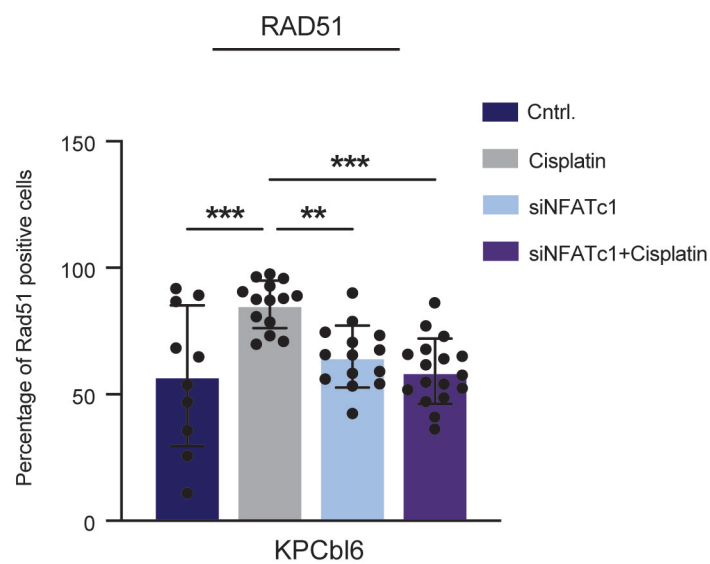**G**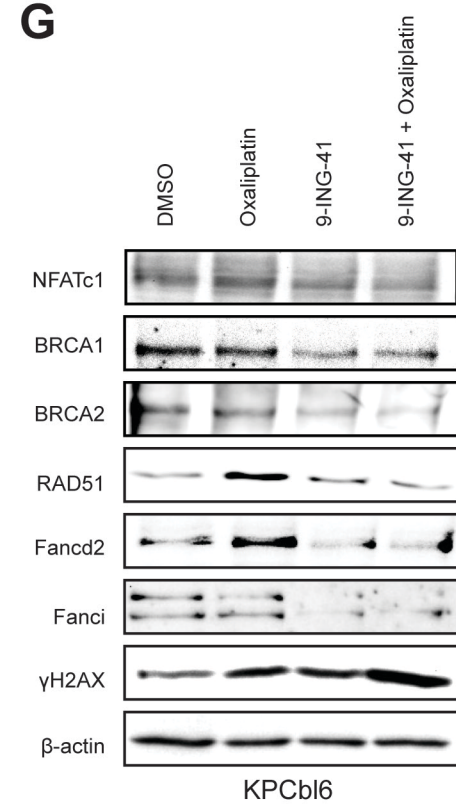

Supplement: online supplemental figure 4 [file gutjnl-75-8-s004.pdf]

**A**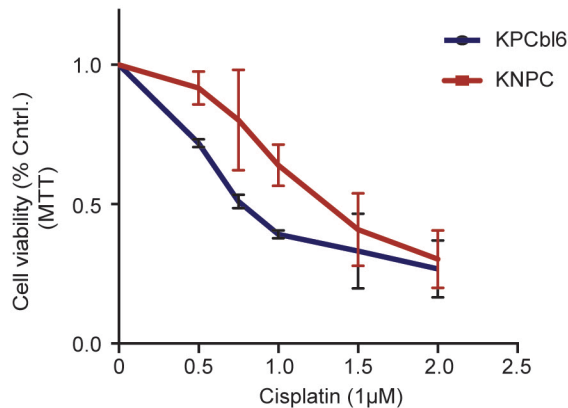**B**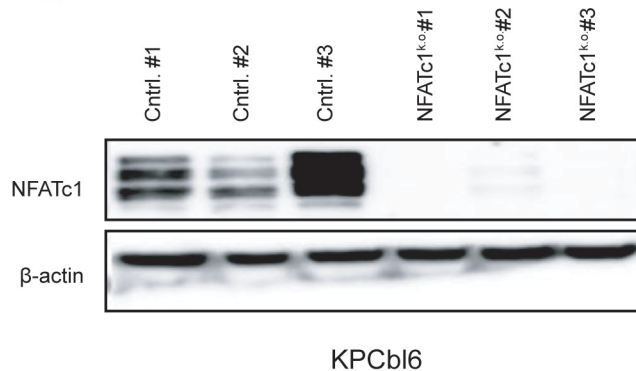**C**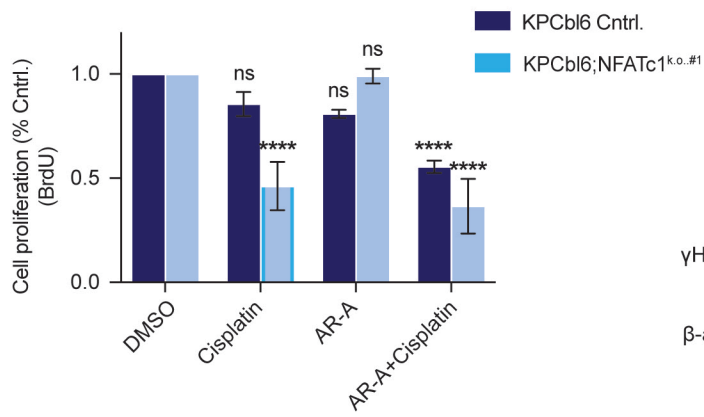**D**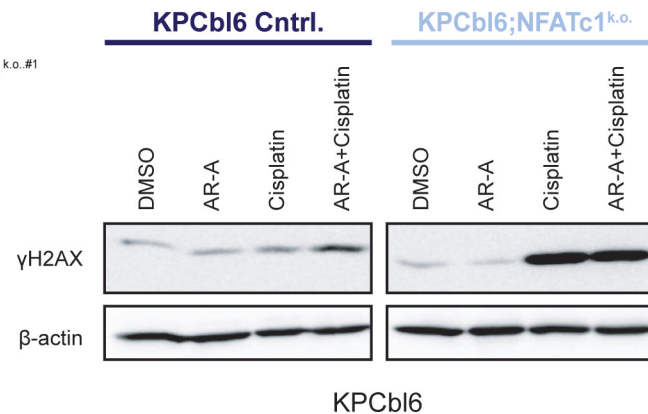

Supplement: online supplemental figure 5 [file gutjnl-75-8-s005.pdf]

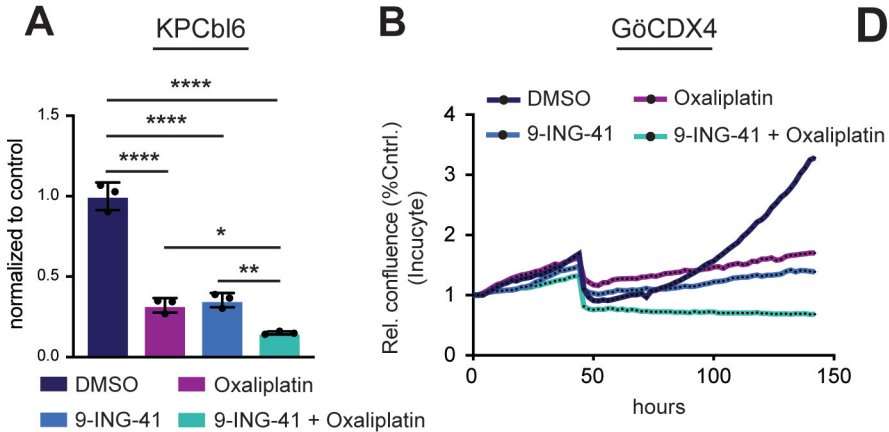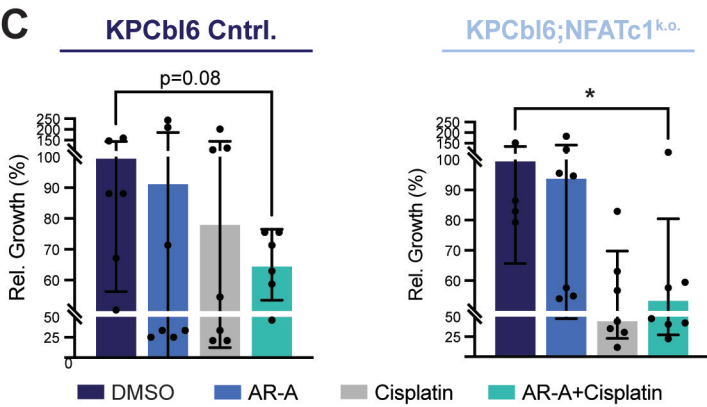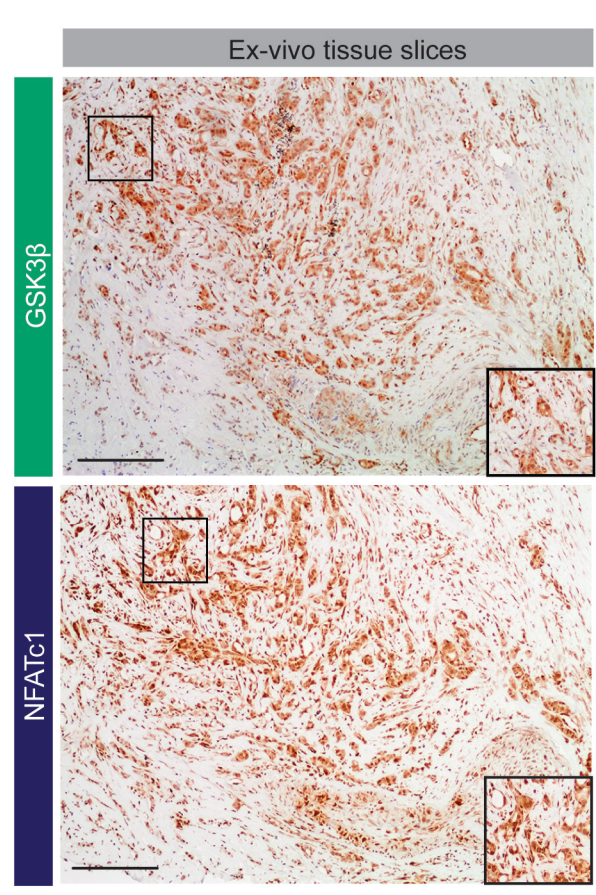

Supplement: online supplemental figure 6 [file gutjnl-75-8-s006.pdf]
