# Supplementary material for: GSK3βhigh/NFATc1high subtype targeting overcomes therapy resistance in pancreatic cancer through transcriptional induction of homologous recombination repair
Source: Gut. 2025 Dec 31;75(8):e336227. doi: 10.1136/gutjnl-2025-336227 (PMC13422050; doi:10.1136/gutjnl-2025-336227)
Supplement: online supplemental file 1 [file gutjnl-75-8-s007.docx]

GSK3β^high^/NFATc1^high^ subtype targeting overcomes therapy resistance in pancreatic cancer through transcriptional induction of homologous recombination repair

Muhammad Umair Latif^1^, Xueang Liu^1^, Aiko Bockelmann^1,9^, Laura Huhnold^1^, Geske Elisabeth Schmidt^1,9^, Lukas Klein^1^, Xueyuan Zhao^1^, Lena Conradi^2,9^, Karly Conrads^3,9^, Anna Lena Weber^1^, Sercan Mercan^1,9^, Kristina Reutlinger^1,9^, Atmika Paul^4,9^, Zeynab Najafova^5^, Steven A. Johnsen^5,12^, Zuriñe Bonilla Del Rio^1^, Frederike Penz^1^, Jovan Todorovic^6,9^, Holger Bastians^4,9^, Tim Beissbarth^3,9^, Ulrich Sax^7,9^, Ramy Ashry^10,11^, Oliver H. Krämer^10^,Elisabeth Hessmann^1,9,13^, Günter Schneider^2,9,13^, Philipp Ströbel^6,9,13^, Ivan Bogeski^8^, Shiv K. Singh^1,9,13^* and Volker Ellenrieder^1,9,13^*

*^1^Department of Gastroenterology, Gastrointestinal Oncology and Endocrinology, University Medical Center Goettingen Germany; ^2^Clinic of General, Visceral and Pediatric Surgery, University Medical Center Goettingen, Germany; ^3^Department of Medical Bioinformatics, University Medical Center Goettingen, Germany; ^4^Department for Molecular Oncology, University Medical Center Goettingen, Germany; ^5^ Robert Bosch Center for Tumor Diseases, Stuttgart, Germany;* ^6^Institute for Pathology, University Medical Center Goettingen, Germany; ^7^Department of Medical Informatics, University Medical Center Goettingen, Germany; ^8^Molecular Physiology, Institute of Cardiovascular Physiology, University Medical Center Goettingen, Germany; ^9^Clinical Research Unit 5002, KFO5002, University Medical Center Goettingen, Germany; ^10^*Institute of Toxicology, University Medical Center, Mainz, Germany; ^11^Department of Oral Pathology, Faculty of Dentistry, Mansoura University, Mansoura, Egypt; ^12^University of Tübingen, Tübingen, Germany. ^13^Comprehensive Cancer Center, Lower Saxony, Göttingen and Hannover, Germany*

*** Authorship Note:** Volker Ellenrieder and Shiv K. Singh are co–senior authors.

**Corresponding author**

Correspondence to Prof. Dr. Volker Ellenrieder, Department of Gastroenterology, Gastrointestinal Oncology and Endocrinology, Robert-Koch Strasse 40, 37075, Goettingen, Germany; [volker.ellenrieder@med.uni-goettingen.de](mailto:volker.ellenrieder@med.uni-goettingen.de).

**Figure Legends**

**Suppl. Fig 1.: A) ﻿**Correlation of GSK3β mRNA expression and survival of patients from TCGA/QCMG/Puleo cohort as depicted in the Kaplan-Meier plot. **B)** Representative IHC images displaying pGSK3β(Tyr216) expression in PDAC patients. Scale bar represents 200µm and 100µm, respectively. **C)** Quantitative analysis of pGSK3β (Tyr216) IHC, n=20. Data are shown as mean±SD. Statistical analysis was performed using Unpaired t-test with Welch’s correction where, ** p≤ 0.01. **D)** Kaplan-Meier plot displaying correlation of nuclear GSK3β expression with patient’s relapse-free survival data. **E)** Kaplan-Meier plot displaying correlation of cytoplasmic GSK3β expression with patient’s survival data. **F)** Kaplan Meier plot displaying correlation of nuclear GSK3α expression with patient’s survival data.  **G)** GöCDX13 and GöCDX26 cells viability was determined using cell titer glo (CellTiter-Glo 3D) under exposure of increasing concentrations of 9-ING-41 (GSK3β-i) for 72 hours. Data are shown as mean±SD. Statistical analysis was performed using One-Way ANOVA where * p≤ 0.05. **H)** GöCDX26 cells proliferation was determined by Incucyte following exposure to 5μM AR-A for up to 8 days. Statistical analysis was performed at day 8 using Unpaired t-test with Welch’s correction where, *** p≤ 0.001. **I)** Representative images IHC, displaying differential GSK3β expression in patient derived organoids. Scale bar indicates 100µm.

**Suppl. Fig 2.: A)** Principal component analysis explaining differential clustering of L3.6pl cells under AR-A and siGSK3β when compared with control cells. **B)** Principal component analysis displaying differential clustering of KPCbl6 cells under AR-A treatment when compared with control cells. **C)** Z_score based heatmap exhibit differential expression of target gene signatures involved in DNA damage repair in KPCbl6 cells. **D)** Validation of BRCA1, BRCA2, RAD51, FANCD2 and FANCI downregulation at mRNA levels following GSK3β inhibition with AR-A for 24 hours in GöCDX13 cells. Data are shown as mean±SD. Statistical analysis was performed using One-Way ANOVA where * p≤ 0.05, ** p≤ 0.01, *** p≤ 0.001 and **** p ≤ 0.0001. **E)** Immunoblots in L3.6pl cells demonstrate dose dependent inactivation of GSK3β along with downregulation of target genes after 48 hours of AR-A treatment. **F)** RT-qPCR validation of target gene signatures in KPCbl6 cells showing downregulation following GSK3β inhibition with 9-ING-41. Data are shown as mean±SD. Statistical analysis was performed using Unpaired t-test with Welch’s correction where ** p≤ 0.01, *** p≤ 0.001 and **** p ≤ 0.0001. **G)** Immunoblots in KPCbl6 cells demonstrate dose dependent inactivation of GSK3β along with downregulation of target gene signatures after 48 hours of 9-ING-41 treatment. **H)** RT-qPCR in GöCDX4 and GöCDX32 displaying downregulation of target genes following siRNA-mediated loss of GSK3β for 48 hours. Data are shown as mean±SD. Statistical analysis was performed using Unpaired t-test with Welch’s correction where ** p≤ 0.01, *** p≤ 0.001 and **** p ≤ 0.0001.

**Suppl. Fig 3.: A-B)** Immunoblot displaying downregulation of NFATc1 expression in GöCDX13 **(A)** and KPCbl6 **(B)** cells after treatment with increasing concentrations of AR-A and 9-ING-41 respectively, for 24 hours. **C)** Schematic description of the workflow of patient’s tissue analysis and data correlation. **D-E)** Identification of clinically relevant cutoff values of **(D)** GSK3β and **(E)** NFATc1 nuclear expression for subtyping.  **F-G)** Kaplan-Meier plots displaying **(F)** overall and **(G)** relapse-free survival of patients with GSK3β^high^/NFATc1^high^ subtype PDAC vs all other individual subtypes. **H-I)** Correlation analysis of NFATc1 expression with patient’s **(H)** overall and (**I)** relapse-free survival. **J-K)** Correlation of GSK3β and NFATc1 expression in PDAC tumor samples with **(J)** Tumor grading and **(K)** tumor size.

**Suppl. Fig 4.: A)** Protein expression of ATR, TopBP1, pCHK1 and CHK1 in GöCDX45 cells following 24 hours treatment with 1µM cisplatin, 5µM AR-A or 1µM 9-ING-41 alone and in combination. **B-D)** Quantitative analysis of mean tail moment (comet) in **(B)** L3.6 cells following treatment with 10µM AR-A, 1µM cisplatin or a combination of both drugs for 24 hours, **(C)** KPCbl6 cells treated with 5µM oxaliplatin, 500nM 9-ING-41 or a combination of both drugs for 24 hours and **(D)** KPCbl6 cells after 24 hours treatment with 1µM cisplatin, siRNA for NFATc1 and combination of both. Data are shown as mean±SD. Statistical analysis was performed using One-Way ANOVA where * p≤ 0.05, ** p≤ 0.01, *** p≤ 0.001 and **** p ≤ 0.0001. **E)** Representative images of RAD51 and γH2AX IF in KCPbl6 cells after NFATc1 silencing alone and combined with cisplatin (1µM) for 24 hours. **F)** Quantitative analysis of RAD51 in KCPbl6 cells with NFATc1 silencing alone and combined with cisplatin (1µM). Data are shown as mean±SD. Statistical analysis was performed using One-Way ANOVA where * p≤ 0.05, ** p≤ 0.01, *** p≤ 0.001, **** p ≤ 0.0001. **G)** Western blot displaying HR genes expression in KPCbl6 cells following 72 hours treatment with 5µM oxaliplatin and 500nM 9-ING-41 alone and in combination.

**Suppl. Fig 5.: A)** MTT assay in KPCbl6 and KNPC cells after treatment for 72 hours with increasing concentrations of cisplatin; n=3. **B)** NFATc1 protein expression in CRISPR/Cas9 KPCbl6 cntrl. and KPCbl6;NFATc1^k.o.^ cells. **﻿** **C) ﻿**BrdU assay was performed in KPCbl6 cntrl. and KPCbl6;NFATc1^k.o.#1^ cells after 48 hours treatment with AR-A and 1µM cisplatin; n=3. Data are shown as mean±SD. Statistical analysis was performed using Two-Way ANOVA where * p≤ 0.05, ** p≤ 0.01, *** p≤ 0.001, **** p ≤ 0.0001. **C)** Induction of DNA damage was confirmed by γH2AX protein expression in KPCbl6 cntrl. and KPCbl6;NFATc1^k.o.#1^ clones following treatment with AR-A alone and in combination with 1µM cisplatin for 24 hours.

**Suppl. Fig 6.: A)** Cell Titer Glo (CTG) assays were conducted in KPCbl6 cells after treatment with 500nM 9-ING-41, 5µM oxaliplatin and combination for 72 hours. **﻿ B)** Incucyte was carried out to determine time-dependent cell proliferation in GöCDX4 cells under treatment with 500nM 9-ING-41, 5µM oxaliplatin and combination. **C)** Relative tumor growth in KPCbl6 cntrl. and KPCbl6;NFATc1^k.o^ mice with respective treatments. **D)** Representative IHC images of GSK3β and NFATc1 IHC in human PDAC explants. Statistical analysis (A, B and C) was performed using One-Way ANOVA where * p≤ 0.05, ** p≤ 0.01 and **** p ≤ 0.0001.

**Material and Methods**

**Human PDAC samples and patient-derived models**

Resected human PDAC tissues for histological analysis and for the development of pre-clinical models (GöCDXs, PDOs and ex vivo explants) were received from the Institute of Pathology and the Department of General, Visceral and Pediatric Surgery of the University Medical Center Göttingen (UMG), Germany. The utilization and characterization of human PDAC data, samples, and models have been approved by the ethical review board of the UMG (11/5/17).

**Animal care, mouse model and treatments**

All animal experiments were approved and carried out according to the regulations of Federation of European Laboratory Animal Science Associations (FELASA, LAVES approval No. 33.33-42502-04-22-00221). Mice breeding was carried out in the Central Animal Facility of the UMG. Mice were kept under controlled atmosphere at ~23±1 °C temperature and 40–60% humidity in 12 hours light and dark cycles with *ad libitum* supply of food and water. Mice were caged separately based upon gender and housed in the same facility. To establish the syngeneic mouse model, pancreata 10-week-old C57BL/6J mice (obtained from Janvier Labs, France) were orthotopically implanted with 3.5×10^4^ KPCbl6 CRISPR/Cas9 control (KPCbl6 Cntrl.) or KPCbl6 CRISPR/Cas9 NFATc1 (KPCbl6;NFATc1^k.o.^) knock-out cells. 9 days after transplantation, the animals were subjected to ultrasound and tumor bearing mice were randomized (following RRR principle) into treatment groups. Mice were treated (i.p.) with GSK3β inhibitor (AR-A 10mg/kg; 5x/week) and cisplatin (4mg/kg; 3x/week) alone or combination of both drugs until day 24. During treatment, body weight and well-being of the animals was recorded 3x/week. Upon treatment termination, tumor size was recorded again via ultrasound and animals were sacrificed. Pancreatic tissues were harvested for molecular and histological analysis. Investigators were kept blind to the allocation of experimental groups and analysis performed. Animal experiments were reported using ARRIVE1 reporting guidelines ^1^. The detailed methods are described in the supplementary file.

### Cell culture, reagents and treatments

For various in vitro studies established human pancreatic cancer cell line L3.6 and ﻿primary murine pancreatic cancer cells isolated from the KPC (KrasLSL.G12D/+; Trp53R172H/+; Pdx-1-Cre) ^2^ and the KNPC (K-rasLSL.G12D/+; ﻿NFATc1c.a.; Trp53R172H/+; P48-Cre/+) ^3^ mouse models respectively, were utilized. In addition, various primary cancer cells (GöCDX, GöCDX4, GöCDX13, GöCDX26, GöCDX32, GöCDX45, GöCDX57, GöCDX84 and GöCDX88) isolated from pancreatic cancer patient derived xenografts (PDX) and Patient-derived organoids (PDO17, PDO53 and PDO61) isolated from primary tumor, as decribed previously^4^, were employed for various molecular, histological and physiological analysis. L3.6 cells were maintained in MEM medium supplemented with 10% FCS. Whereas KPC and KNPC cells were maintained in DMEM medum supplemented with 10% FCS and 1% nonessential MEM amino acids. GöCDX cells were maintained in RPMI medium supplemented with 10% FCS. Cells were always plated overnight before the start of any treatment. PDOs were maintained and cultivated in conditioned media as described before ^4^. HCT116 (human colorectal cancer) cells were obtained from ATCC (USA). The cells were cultivated in RPMI1640 medium (PAN-Biotech GmbH, Germany), supplemented with 10% fetal bovine serum (FBS; Corning Inc., USA), 100 units/mL penicillin, and 100 μg/mL streptomycin (Anprotec, Germany). GSK3β Inhibitors 9-Ing41 (Aobious, Gloucester, MA, USA) and AR-A014418 (Abmole Bioscience Inc., Houston, USA) were dissolved in DMSO. Cisplatin (Merck, Darmstadt, Germany) was dissolved in water. All the cell culture experiments were performed in technical and biological triplicates.

﻿**CRISPR/Cas9-mediated NFATc1 knockout**

NFATc1 stable knock-out in KPCbl6 cells was accomplished by CRISPR/Cas9 mediated gene editing as decsribed previously ^4,5^. In brief, according to the gene sequence obtained from NCBI database, appropriate Single guide RNAs (sgRNAs) targeting exon 3 were desigend and constructed. The guide RNAs were cloned into a pSpCas9(BB)-2A-GFP (PX458) plasmid containing Cas9 endonuclease, a green fluorescent protein (GFP)-tag for sorting purposes and an ampicillin resistance for selection. Plasmid was transfected into the cells using OptiMEM (﻿Thermo Fischer Scientific, Waltham, USA) and 25µL of Lipofectamin 2000 (﻿Thermo Fischer Scientific, Waltham, USA). After 48 h after transfection cells were trypsinized and sorted as single cell clones based on GFP-positivity. The single cell clones were expanded and selected for NFATc1 knockout. NFATc1 knockout was confirmed by Western blot and RT-qPCR. Clones were also sequenced (Microsynth Seqlab, Göttingen) to confirm the knockout of NFATc1 in KPCbl6 cells. ﻿Oligonucleotides sequences used are provided in table 1. HCT116 + DRGFP stable cells were generated by transfecting HCT116 cells with 2 μg pHPRT-DRGFP^6^ (Addgene #26476), using Lipofectamine™ 3000 transfection reagent (Thermo Fisher, Scientific, USA), according to the manufacturer’s instructions. After 48 hours of transfection, selection was carried out with 1 μg/mL puromycin (Santa Cruz, USA). Stable single cell clones were validated by DRGFP assay ^7^ and maintained in medium supplemented with 1 μg/mL puromycin. All cells were grown in a humidified incubator with an atmosphere of 37°C and 5% CO_2_.

**siRNA and Plasmid Transfection**

KPCbl6 cells were transfected for required time points with murine NFATc1 (Thermo fischer scientific 288360) and GSK3β (Thermo fischer scientific 185671) siRNA. L3.6 cells were transfected with human GSK3β (Thermo fischer scientific S6240) siRNA as described before ^6,7^. Briefly, Cells were seeded and allowed to grow until 60% confluency. siRNA constructs were prepared in ﻿500 µL of Opti-MEM (Thermo Fisher Scientific, Waltham, Massachusetts, USA) mixed with 6µL siRNA and 6µL of siLentFect lipid reagent (Bio-Rad; 170-3362). The mix was incubated for 20 min at RT and then supplemented to the normal growth media. In CDX cell lines siRNA-mediated knockdown of GSK3B and NFATc1 was achieved using reverse transfection as described before ^6^. KPCbl6 cells were transfected with constitutively active NFATc1 (HA-tagged MSCV, Amp) and GSK3β (wt HA-tagged pcDNA3, Amp) constructs. Briefly, cells were seeded in a 6-well plate until the required confluency. Transfection mixture was prepared in 500 µL of Opti-MEM with 1 µg of the needed plasmid and Lipofectamine2000. After 10 minutes incubation at room temperature, mix was added to the cells along with 500µL of serum containing media.

**Cell viability**

Cells viability was assesed via MTT (3-[4,5-dimethylthiazol-2-yl]-2,5 diphenyl tetrazolium bromide) assay (﻿Biomol, Hamburg, Germany), CellTiter-Glo® Cell Viability Assay (Promega) and Crystal violett assay (﻿0.1% (w/v) crystal violett, 20% (v/v) EtOH in H_2_O) in PDAC cell lines, GöCDX cells and PDAC organoids (PDOs). Briefly, cells were seeded in 24 or 96-well plates for overnight prioir to respective treatment. After treatment cells were incubated with MTT or cell titer glo solution as described by the manufacturer. ﻿The amount of metabolized MTT was measured at 595nm using photometer and cell titer glo at 570nm using luminometer, respectively. For crystal violet staining KPCbl6 cells following treatment were incubated for 10 minutes covered in crystal violet solution. After the removal of the solution, cells were washed with H_2_O to remove unbound crystal violett. Staining was documented by pictures and quantified by dissolving in MTT solubilization solution and measurement at 595 nm with a photometer.

**Cell proliferation**

Tumor cell proliferation was examined using bromodeoxyuridine (BrdU, colorimetric 11647229001, Merck, Darmstadt, Germany) Labeling. Briefly, cells were seeded in 96-wells, overnight prior to treatment. Cell proliferation was estimated following manufacturer's protocol of the colorimetric cell proliferation ELISA kit. Patient-derived xenograft (CDX) cells were seeded in 96-well plates with a cell count showing linear growth rates. After 48 hours, cells were treated with indicated concentrations of respected drugs at 37°C and 5% CO_2_ for up to 7 days. Cell confluence was measured using IncuCyte® S3 (Sartorius AG, Goettingen, Deutschland) and analyzed with Incucyte Base Software (2020B, Sartorius AG, Goettingen, Deutschland). The confluence at day 5 was normalized to the confluence at day 0 for each well and normalized to the treatment control.

﻿**Flow cytometry (Cell cycle)**

Cell cycle analysis in L3.6, KPCbl6 and CDX cells was performed using flow cytometry. At the end of corresponding treatment endpoint, cells were trypsinized and same amount of the cells were fixed by centrifugation at 1200 rpm for 3 minutes at room temperature (RT) followed by resuspension of the pellet in 600µL PBS. The samples were vortexed and 1.4 mL of the ice-cold absolute ethanol was added dropwise. After incubation for 30 min at 4 °C, samples were centrifuged at 1,200 rpm, 3 minutes, RT. After discarding the supernatant, pellet was washed twice with PBS supplemented with RNase A (5 µg/mL). Cells were then resuspended in 100µL of PBS containing 5µL of Hoechst. The samples were incubated for 30 minutes at 37 °C and diluted with 400 µL of PBS before measurements (FACS CantoII, BD Biosciences). Data was processed using FlowJo software.

**Chromatin Immunoprecipitation (ChIP)**

### For the analysis of NFATc1 binding on the target gene signatures, Chromatin Immunoprecipitation (ChIP) analysis was performed as described previously ^4^. KPCbl6 cells were subjected to either GSK3β inhibition (AR-A) for 24 hours or treatment with vehicle control followed by lysate preparation and precipitation of IgG and NFATc1 antibody. Later, RT-qPCR was performed in triplicates using primers listed in supplementary table 2. PCR results were normalized to input and visualized and statistically analyzed by GraphPad Prism 9 software.

**HR Repair assay**

The HR repair assay was performed essentially as described before ^8^. Briefly, HCT116-DRGFP cells were seeded in 6 well plates to a confluency of 80% and transfected with siRNAs against either *BRCA1*, GSK3β, NFATc1 or a non-targeting control (30–90 pmol; Sigma-Aldrich, Germany) using Lipofectamine™ RNAiMAX (Thermo Fisher Scientific, USA), according to the manufacturer’s protocols. After 6 hours, these cells were transfected with 2 µg of expression constructs for either pcDNA3.1 empty vector control or I-SceI endonuclease expression vector, pCBA-SceI ^9^ (Addgene #26477) by Lipofectamine™ 3000. After 18 hours of transfection, media was exchanged with fresh media and cells were treated with DMSO or 10 µM GSK3βi for 24 hours. The cells were harvested, resuspended with PBS and the levels of GFP expression was measured by flow cytometry in the BD FACSCanto II (BD Biosciences) using the BD FACSDiva Software (BD Biosciences). The data is presented as the mean±SD value in three biologically replicates.

﻿**RNA isolation and real-time PCR (RT-qPCR)**

RNA was isolated using using phenol-chloroform purification via TRIzol (Invitrogen) according to manufacturer’s instructions ^7^. After estimating RNA quality and concentration cDNA was synthesized using iScript cDNA Synthesis Kit (170-8891, BioRad,) as described before ^7^. RT-qPCR for mRNA expression analysis was performed in triplicates using iTaq Universal SYBR Green Supermix (BioRad, 172-5125) with StepOne Plus Real-Time PCR System (Applied Biosystems). mRNA expression of target genes was normalized to housekeeping gene. List of all the primer sequences is provided in Supplementary Table 3.

﻿**RNA sequencing**

Whole transcriptome analysis was performed in L3.6 and KPCbl6 cells as described before ^4^. L3.6 cells were subjected to either GSK3β inhibition with AR-A 014418 (10µM) or knock-down of GSK3β for 48 hours. KPCbl6 cells were simply treated with AR-A 014418 (10µM) for 48 hours. RNA was isolated using Trizol and its quality was checked via agarose gel electrophoresis. cDNA libraries were prepared using the TruSeq RNA Library Prep Kits (RS-122-2001; RS-122-2002) following the manufacturer's protocol. cDNA concentrations were measured via Qubit (Thermo Scientific, Q32854). Further, the fragment sizes and purity were estimated by using the Agilent Bioanalyzer 2100 (high sensitivity DNA analysis kit, Agilent 5067-4626). Samples were pooled and sequenced by the NGS Integrative Genomics Core Unit, University Medical Center Göttingen, Germany.

Raw sequencing reads were quality checked using FastQC v0.11.4 (https://www. bioinformatics.babraham.ac.uk/projects/fastqc/). Alignment was performed by STAR v2.7.3a ^10^ against reference genome hg38 with GENCODE v38 gene annotation. Aligned reads were sorted and indexed using samtools v1.9 ^11^ and assigned to genes using “htseq-count” function of HTSeq v0.11.3 ^12^. Downstream analysis of count files was performed in R v4.2.0. Differential expression was performed using DESeq2 v1.36.0 ^13^. Heatmaps were generated using the R package pheatmap v1.0.12. GSEA and GO term analysis was performed using the clusterProfiler package v4.4.4 ^14^ using “GSEA” and “enricher” functions with gene sets of the Molecular Signature Database ^15^.

**Immunohistochemistry and Immunofluorescence**

Hematoxylin eosin staining and IHC were performed on paraffin fixed human and murine PDAC tumor sections as well as patient derived organoids and ex-vivo tissue slices, using standard protocol as previously described ^7^. The antibodies used are listed in supplementary table 4 and 5. For immunofluorescence, cells were fixed with 4% (v/v) paraformaldehyde (PFA). After washing cells were permeabilized by incubation with 10% normal goat serum (NGS) in phosphate-buffered (PB) solution supplemented with Triton X-100 for one hour followed by overnight incubation with primary antibodies. After washing steps, cells were incubated with secondary antibody for one hour. Subsequently, cells were counterstained with DAPI and mounted with coverslips. Staining was analyzed using fluorescence microscope software. For immunostaining and immunofluorescence, scale bar in the images are 100µm and 200µm respectively, unless otherwise indicated. The percentage of positive tumor cells against respective antibodies were quantified using QuPath ^16^.

**Clinical patient analysis**

The percentage of positive tumor cells for GSK3B and NFATc1 was determined using QuPath on whole slide images of respective IHC stainings of patient tumor tissue. Cutoffs for separation of GSK3Bhigh/low and NFATc1high/low status was determined using the R2 platform (R2 Genomics Analysis and Visualization Platform (<http://r2.amc.nl)>). Survival analysis was performed in R v4.2.0 using the survival v3.5-8, ggsurvfit v1.0.0, and survminer v0.4.9. Significance was determined using log-rank test. Correlation analysis was performed using ggpubr v0.6.0 and ggplot2 v3.5.1, showing Spearman’s *R* and associated *P* value for correlations, as well as linear regression with 95% confidence interval. Full clinical patient information is shown in Supplementary Table 7.

﻿**Immunoblot**

Protein expression of target genes was analyzed by western blot as described previously ^6,7^. Briefly, at the end of respective treatments, cells were harvested in lysis buffer containing protease inhibitors (Roche; 11697498001). Protein concentrations were estimated using Bradford assay. Equal amounts of proteins were loaded and resolved via 10/15% (v/v) SDS–PAGE followed by transfer to nitrocellulose membranes via semi-dry blotting system (Bio-Rad; 10026938). Membranes were washed with TBST and blocked in 5% (w/v) milk or BSA powder in TBST for 1 hour at RT. Primary antibodies were added for overnight at 4 °C. After washing, membranes were incubated with Secondary HRP-linked antibodies for 1 hour at RT. Proteins were detected using ECL substrate (Bio-Rad) in Intas ChemoCam Imager and analyzed using ChemoStar Software. Antibodies used in immunoblotting are listed in Supplementary Table 6.

**Alkaline Comet (Single-Cell Gel Electrophoresis) Assay**

Treatment of L3.6 and KPCbl6 cells with 1μΜ cisplatin ± 10μΜ AR-A for 24 hours was employed. Positive control cells were treated with 300 µM tert-Butyl hydroperoxide (TBHP) (Sigma-Aldrich, Taufkirchen, Germany) for 2 hours. Alkaline comet assay was performed as previously reported ^17^**.** Tail moment depicting DNA damage was assessed, and exemplary images were captured with Comet IV software (Perceptive Imaging, Liverpool, U.K.). At least 200 cells were measured for each sample per experiment. In another approach KPCbl6 cells were treated with 5μΜ oxaliplatin ± 500nM 9-ING-41 and 1μΜ cisplatin ± siRNA for NFATc1 for 24 hours and subjected to similar procedure.

**Ex-vivo tumor slices and generation of human PDAC explants**

PDAC tumor slices were prepared and cultured ex vivo as described before ^18^. Briefly, absorbable haemostatic gelatine sponges (CuraMedical) were prepared before the tumor resection. Small sections of freshly resected human PDAC tissues were embedded in agarose and sectioned into ~300 μm thick slices with vibratome (Leica VT 1200S). The slices were then cultured in 24 well tissue culture plates containing gelatin sponges and optimized media as described before^18^. The tissue slices were subjected to 48 hours of 2.5μΜ cisplatin ± 5μΜ AR-A treatment. Cell growth in the tissue slices were assessed by CTG. At the end of treatment timepoint slices were embedded into paraffin and utilized for histological analysis i.e., HE staining and IHC for γH2AX, RAD51 and BRCA1.

### Statistical analysis

Data are expressed as mean±SD until unless mentioned otherwise. Statistical analysis was perfomed by Graphpad Prism 9.0 using Unpaired t-test, One-Way ANOVA and Two-Way ANOVA, respectively (described in each figure legend). Grubb's test was used to identify the statistically significant outlier. Statistical significance was always mentioned as **p* < 0.05, ***p* <0.005, ****p* < 0.0005, *****p*<0.0001.

Table 1. Oligonucleotides for generation of NFATc1 knockout cells in murine KPCbl6 cells

| Target | Sequence |
| --- | --- |
| ﻿NFATc1 Exon3 | ﻿5'GACCGGCTGTAGCTCGGCACTGCAG |
| ﻿NFATc1 Exon3 | ﻿5'AAACCTGCAGTGCCGAGCTACAGCC |

Table 2. Primer sequences used for ChIP experiment

| Name | Sequence for | Sequence rev |
| --- | --- | --- |
| M Brca1-E1-2 | GCT GGT AGT CTG GCC TGA TG | GTT ACG TAT CCT GGG CTC GC |
| M Brca2 | TGC ACT GGG CTT GTT ACT CA | GCG ATC ACC GTA TTT CCC GA |
| M Rad51 Prom. | ACA TCC GCT GTG CAA GAA GT | TCA TCA CGC CTT TCT CCG AC |

Table 3. Primer sequences used RT-qPCR

| Name | Sequence for | Sequence rev |
| --- | --- | --- |
| h GSK3β | CGA GAC ACA CCT GCA CTC TT | TTA GCA TCT GAC GCT GCT GT |
| h BRCA1 | GCCTTCTAACAGCTACCCTTC | CTTCTGGATTCTGGCTTATAGGG |
| h BRCA2 | TGCTGGGTAGATGCAGACAC | AGCCATTCCCTCTGTGAGGA |
| h Rad51 | TGC GGA CCG AGT AAT GGC | CCA CAC TGC TCT AAC CGT GA |
| h Fancd2 | AA GTC GAA AAC TAC GGG CGG | ATG AGG AAG CCA AGG TTC GG |
| h Fanci | GGTT GAA GTA GGG GAC AGG T | ATA AGG TGC CCT GTG TCC AG |
|  |  |  |
| m Gsk3β | AGG AAC ACC AAC AAG GGA GC | TCC TGG GGT GAA ATG TCC TG |
| m Brca1 | GGA TCC AGC ACC TCT CTT GG | TGT AAG CTG CAT TCC CGT GT |
| m Brca2 | GTC TGG GAG TTG AAG TGG ATC | CAA AGT AGC GGG AGA GTC AG |
| m Rad51 | GAT ACG GTC TCT CTG GCA GC | GCT TGG TAA AGG AGC TGG GT |
| m Fancd2 | CTC GAG AAG ACG GTG AAG GG | AAG CTG AAG TCG AGG AGT GG |
| m Fanci | ATC TCG CTG TTG TGC TTG GA | GAC CTC TGA AAC TGC CGG AT |
| RPLP0 (XS13) human/ mouse | TGG GCA AGA ACA CCA TGA TG | AGT TTC TCC AGA GCT GGG TTG T |

Table 4. Antibodies used for IHC

| Name | Company | Article number |
| --- | --- | --- |
| NFATc1 | abcam | ab25916 |
| GSK3β | CellSignaling | 12456 |
| γH2AX | CellSignaling | 80312 |
| RAD51 | abcam | ab133534 |
| BRCA1 | Santa Cruz | sc-135732 |

Table 5. Antibodies used for IF

| Name | Company | Article number |
| --- | --- | --- |
| γH2AX | CellSignaling | 80312 |
| BRCA1 | Santa Cruz | sc-135732 |
| Rad51 | abcam | ab133534 |

Table 6. Antibodies used for WB

| Name | Company | Article number |
| --- | --- | --- |
| β-actin HRP-linked | Sigma | A3854 |
| Phospho-Glycogensynthase | CellSignaling | 3891 |
| BRCA1 | Genetex | GTX70111 |
| BRCA2 | abcam | ab27976 |
| Rad51 | Santa Cruz | sc-53428 |
| Fancd2 | abcam | Ab108928 |
| GSK3beta | CellSignaling | 9315 |
| γH2AX | CellSignaling | 80312 |
| HA-Tag | CellSignaling | 2367 |
| NFATc1 | Santa Cruz | Sc-7294 |
| ATM | abcam | Ab78 |
| ATR | CellSignaling | 13934S |
| p-Chk1 (Ser345) | CellSignaling | 2348 |
| CHK1 | CellSignaling | 2345 |

Table 7. PDAC patients clinical data

**References:**

1. du Sert NP, Ahluwalia A, Alam S, et al. Reporting animal research: Explanation and elaboration for the arrive guidelines 2.0. *PLoS Biol*. 2020;18(7). doi:10.1371/journal.pbio.3000411

2. Hingorani SR, Wang L, Multani AS, et al. Trp53R172H and KrasG12D cooperate to promote chromosomal instability and widely metastatic pancreatic ductal adenocarcinoma in mice. *Cancer Cell*. 2005;7(5). doi:10.1016/j.ccr.2005.04.023

3. Singh SK, Chen N, Hessmann E, et al. Antithetical NFAT c1–Sox2 and p53–miR200 signaling networks govern pancreatic cancer cell plasticity . *EMBO J*. 2015;34(4). doi:10.15252/embj.201489574

4. Hasselluhn MC, Schlösser D, Versemann L, et al. An NFATc1/SMAD3/cJUN Complex Restricted to SMAD4-Deficient Pancreatic Cancer Guides Rational Therapies. *Gastroenterology*. 2024;166(2):298-312.e14. doi:10.1053/J.GASTRO.2023.10.026

5. Sen M, Wang X, Hamdan FH, et al. ARID1A facilitates KRAS signaling-regulated enhancer activity in an AP1-dependent manner in colorectal cancer cells. *Clin Epigenetics*. 2019;11(1). doi:10.1186/s13148-019-0690-5

6. Tu M, Klein L, Espinet E, et al. TNF-α-producing macrophages determine subtype identity and prognosis via AP1 enhancer reprogramming in pancreatic cancer. *Nat Cancer*. 2021;2(11):1185-1203. doi:10.1038/s43018-021-00258-w

7. Latif MU, Schmidt GE, Mercan S, et al. NFATc1 signaling drives chronic ER stress responses to promote NAFLD progression. *Gut*. 2022;71(12):2561-2573. doi:10.1136/GUTJNL-2021-325013

8. Pierce AJ, Hu P, Han M, Ellis N, Jasin M. Ku DNA end-binding protein modulates homologous repair of double-strand breaks in mammalian cells. *Genes Dev*. 2001;15(24). doi:10.1101/gad.946401

9. Richardson C, Moynahan ME, Jasin M. Double-strand break repair by interchromosomal recombination: Suppression of chromosomal translocations. *Genes Dev*. 1998;12(24). doi:10.1101/gad.12.24.3831

10. Dobin A, Davis CA, Schlesinger F, et al. STAR: Ultrafast universal RNA-seq aligner. *Bioinformatics*. 2013;29(1). doi:10.1093/bioinformatics/bts635

11. Danecek P, Bonfield JK, Liddle J, et al. Twelve years of SAMtools and BCFtools. *Gigascience*. 2021;10(2). doi:10.1093/gigascience/giab008

12. Anders S, Pyl PT, Huber W. HTSeq-A Python framework to work with high-throughput sequencing data. *Bioinformatics*. 2015;31(2). doi:10.1093/bioinformatics/btu638

13. Love MI, Huber W, Anders S. Moderated estimation of fold change and dispersion for RNA-seq data with DESeq2. *Genome Biol*. 2014;15(12). doi:10.1186/s13059-014-0550-8

14. Yu G, Wang LG, Han Y, He QY. ClusterProfiler: An R package for comparing biological themes among gene clusters. *OMICS*. 2012;16(5). doi:10.1089/omi.2011.0118

15. Liberzon A, Birger C, Thorvaldsdóttir H, Ghandi M, Mesirov JP, Tamayo P. The Molecular Signatures Database Hallmark Gene Set Collection. *Cell Syst*. 2015;1(6). doi:10.1016/j.cels.2015.12.004

16. Bankhead P, Loughrey MB, Fernández JA, et al. QuPath: Open source software for digital pathology image analysis. *Sci Rep*. 2017;7(1). doi:10.1038/s41598-017-17204-5

17. Pons M, Nagel G, Zeyn Y, et al. Human platelet lysate as validated replacement for animal serum to assess chemosensitivity. *ALTEX*. 2019;36(2). doi:10.14573/altex.1809211

18. Decker-Farrell AR, Ma A, Li F, Muir A, Olive KP. Generation and ex vivo culture of murine and human pancreatic ductal adenocarcinoma tissue slice explants. *STAR Protoc*. 2023;4(4):102711. doi:10.1016/j.xpro.2023.102711
