# Supplementary material for: GSK3βhigh/NFATc1high subtype targeting overcomes therapy resistance in pancreatic cancer through transcriptional induction of homologous recombination repair
Source: Gut. 2025 Dec 31;75(8):e336227. doi: 10.1136/gutjnl-2025-336227 (PMC13422050; doi:10.1136/gutjnl-2025-336227)
Supplement: online supplemental file 2 [file gutjnl-75-8-s008.pptx]

## Slide 1
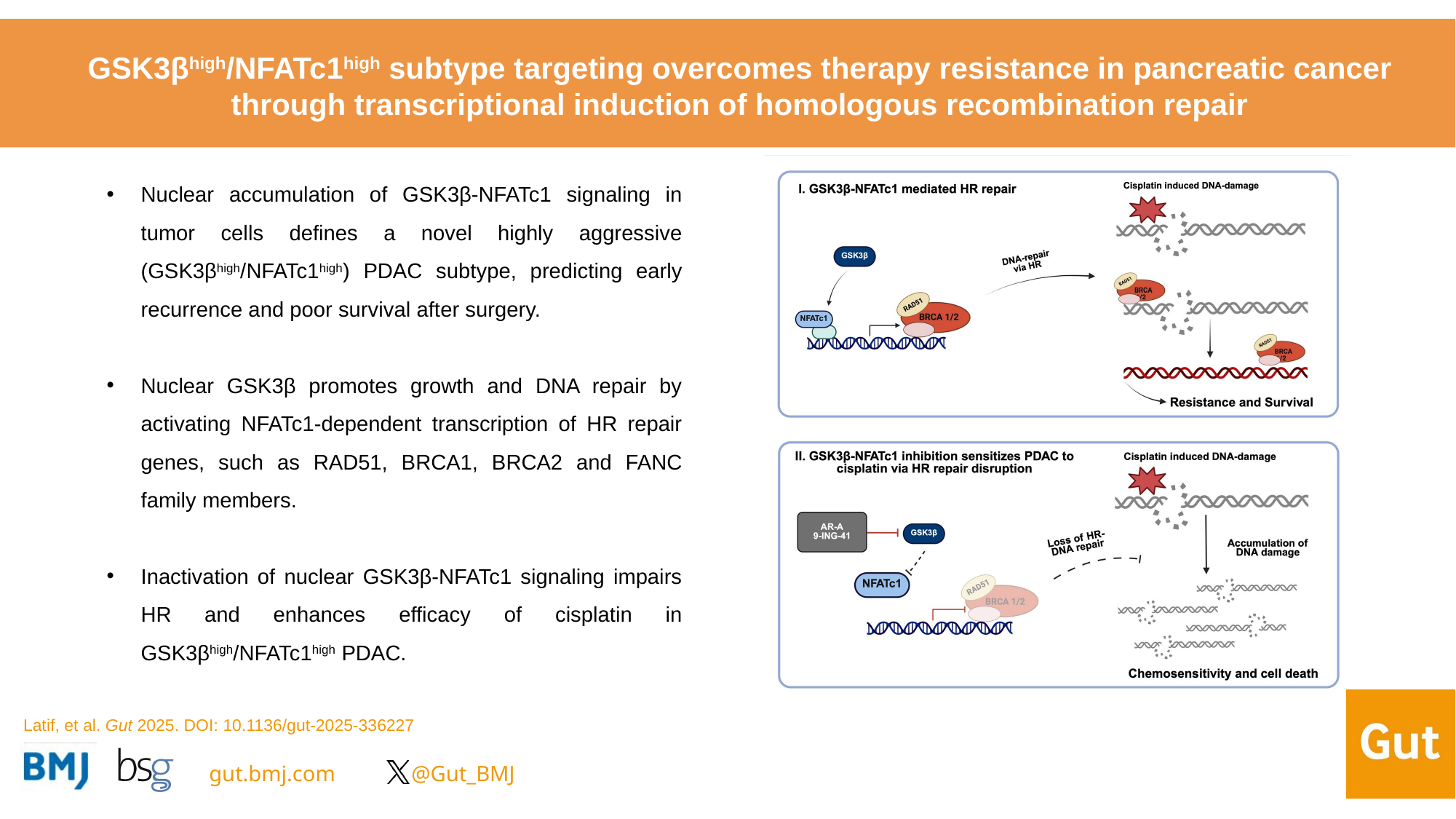

GSK3βhigh/NFATc1high subtype targeting overcomes therapy resistance in pancreatic cancer through transcriptional induction of homologous recombination repair
Nuclear accumulation of GSK3β-NFATc1 signaling in tumor cells defines a novel highly aggressive (GSK3βhigh/NFATc1high) PDAC subtype, predicting early recurrence and poor survival after surgery.
Nuclear GSK3β promotes growth and DNA repair by activating NFATc1-dependent transcription of HR repair genes, such as RAD51, BRCA1, BRCA2 and FANC family members.
Inactivation of nuclear GSK3β-NFATc1 signaling impairs HR and enhances efficacy of cisplatin in GSK3βhigh/NFATc1high PDAC.
Latif, et al. Gut 2025. DOI: 10.1136/gut-2025-336227
gut.bmj.com
@Gut_BMJ
